# Neutralization Takes Precedence Over IgG or IgA Isotype-related Functions in Mucosal HIV-1 Antibody-mediated Protection

**DOI:** 10.1016/j.ebiom.2016.11.024

**Published:** 2016-11-21

**Authors:** Rena D. Astronomo, Sampa Santra, Lamar Ballweber-Fleming, Katharine G. Westerberg, Linh Mach, Tiffany Hensley-McBain, Laura Sutherland, Benjamin Mildenberg, Georgeanna Morton, Nicole L. Yates, Gregory J. Mize, Justin Pollara, Florian Hladik, Christina Ochsenbauer, Thomas N. Denny, Ranjit Warrier, Supachai Rerks-Ngarm, Punnee Pitisuttithum, Sorachai Nitayapan, Jaranit Kaewkungwal, Guido Ferrari, George M. Shaw, Shi-Mao Xia, Hua-Xin Liao, David C. Montefiori, Georgia D. Tomaras, Barton F. Haynes, M. Juliana McElrath

**Affiliations:** a Vaccine and Infectious Disease Division, Fred Hutchinson Cancer Research Center, Seattle, WA, USA; b Center of Virology and Vaccine Research, Beth Israel Deaconess Medical Center, Boston, MA, USA; c Duke Human Vaccine Institute, Duke School of Medicine, Durham, NC, USA; d Department of Obstetrics and Gynecology, University of Washington, Seattle, WA, USA; e Department of Medicine, University of Alabama at Birmingham, Birmingham, AL, USA; f Department of Medicine, Perelman School of Medicine, University of Pennsylvania, Philadelphia, PA, USA; g Department of Disease Control, Ministry of Public Health, Nonthaburi, Thailand; h Department of Clinical Tropical Medicine, Mahidol University, Bangkok, Thailand; i Royal Thai Army Component, Armed Forces Research Institute of Medical Sciences (AFRIMS), Bangkok, Thailand; j Department of Tropical Hygiene, Mahidol University, Bangkok, Thailand; k Department of Medicine, University of Washington, Seattle, WA, USA; l Department of Laboratory Medicine, University of Washington, Seattle, WA, USA; m Department of Global Health, University of Washington, Seattle, WA, USA

**Keywords:** ADCC, Antibody-dependent cell-mediated cytotoxicity, ARV, Anti-retroviral, AZT, Zidovudine, bnAb, Broadly neutralizing antibody, CD4bs, CD4 binding site, dIgA, Dimeric IgA, FcRn, Neonatal Fc receptor, GalCer, Galactosyl ceramide, GI, Gastrointestinal, GU, Genitourinary, IDV, Indinivir, IMC, Infectious molecular clone, LED, Lowest effective dose, mAb, Monoclonal antibody, mIgA, Monomeric IgA, MPER, Membrane-proximal external region, nnAb, Non-neutralizing antibody, pIgR, Polymeric IgA receptor, SC, Secretory component, snLuc, Secreted nanoluciferase, sIgA, Secretory IgA, T/F, Transmitted/founder, Antibodies, Neutralizing antibodies, HIV-1, Mucosal immunology, Non-human primate rectal challenge model, Vaginal explants, IgA, IgG

## Abstract

HIV-1 infection occurs primarily through mucosal transmission. Application of biologically relevant mucosal models can advance understanding of the functional properties of antibodies that mediate HIV protection, thereby guiding antibody-based vaccine development. Here, we employed a human *ex vivo* vaginal HIV-1 infection model and a rhesus macaque *in vivo* intrarectal SHIV challenge model to probe the protective capacity of monoclonal broadly-neutralizing (bnAb) and non-neutralizing Abs (nnAbs) that were functionally modified by isotype switching. For human vaginal explants, we developed a replication-competent, secreted NanoLuc reporter virus system and showed that CD4 binding site bnAbs b12 IgG1 and CH31 IgG1 and IgA2 isoforms potently blocked HIV-1_JR-CSF_ and HIV-1_Bal26_ infection. However, IgG1 and IgA nnAbs, either alone or together, did not inhibit infection despite the presence of FcR-expressing effector cells in the tissue. In macaques, the CH31 IgG1 and IgA2 isoforms infused before high-dose SHIV challenge were completely to partially protective, respectively, while nnAbs (CH54 IgG1 and CH38 mIgA2) were non-protective. Importantly, in both mucosal models IgG1 isotype bnAbs were more protective than the IgA2 isotypes, attributable in part to greater neutralization activity of the IgG1 variants. These findings underscore the importance of potent bnAb induction as a primary goal of HIV-1 vaccine development.

## Highlights

• Only broadly-neutralizing IgG and IgA Abs were protective in two relevant mucosal models of HIV-1 infection.
• IgG isotype variants were typically more protective than the IgA isotype variants against vaginal or rectal challenge.
• Neutralization rather than IgA-specific functions may afford better protection against mucosal HIV-1 infection.

Antibodies are likely to play a key role in preventing HIV-1 infection following mucosal exposure. We used two mucosal models, in conjunction with IgG and IgA versions of the same antibodies, to identify protective antibody properties relevant to vaginal and rectal transmission. Antibodies that can potently neutralize most HIV-1 strains were protective against viral challenge, but those without this capability were non-protective. The IgA antibodies were no more protective than their IgG counterparts even though IgA antibody forms are more abundant in the mucosa. These findings can help prioritize vaccine designs to those that induce potent broadly neutralizing IgG antibodies.

## 1 Introduction

HIV-1 transmission occurs primarily through virus infection of genitourinary (GU) or gastrointestinal (GI) mucosae following sexual exposure (UNAIDS, 2014a, UNAIDS, 2014b, WHO in partnership with UNICEF and UNAIDS, 2014). Biomedical interventions that can act at portals of HIV-1 entry may be most effective at preventing infection, thwarting the initial seeding of the viral reservoir and limiting systemic spread. Successful immune-based approaches will likely require potent vaccine-induced antibodies (Abs) or passively administered Abs that, in concert with local immune responses, can rapidly defend against mucosal infection. Thus, understanding the properties and functions of Ab-mediated protection against mucosal HIV-1 infection is key in designing effective, multi-layered defense strategies against mucosal transmission of HIV-1.

The potential protective role of broadly reactive, potent anti-Env neutralizing Abs (bnAbs) against HIV-1 infection at mucosal sites has been recognized for decades (Klein et al., 2013a, Moldt et al., 2012, Shingai et al., 2014, Liu et al., 2016, Hessell et al., 2009b, Barouch et al., 2013, Pegu et al., 2014, Stamatatos et al., 2009, Mascola and Haynes, 2013, Hessell et al., 2009a, Mascola et al., 2000, Veazey et al., 2003), but as yet no candidate vaccine tested has elicited these Abs in the circulation of HIV-1-uninfected persons. By contrast, broadly neutralizing Abs (bnAbs) emerge in about 50% of HIV-infected persons within two years of infection (Stamatatos et al., 2009, Hraber et al., 2014), and potent human monoclonal Abs (mAbs) of various epitope specificities that are capable of neutralizing > 90% of circulating HIV-1 strains have been generated from memory B cells of HIV-infected persons (Mascola and Haynes, 2013, Burton and Mascola, 2015, Walker et al., 2011, Falkowska et al., 2012, Wu et al., 2010). These bnAbs, delivered as passive immunoprophylaxis, are undergoing clinical evaluation for prevention against HIV-1 acquisition (Caskey et al., 2015, Ledgerwood et al., 2015) after promising results in non-human primate (NHP) studies (Klein et al., 2013a, Pegu et al., 2014, Shingai et al., 2014). Findings in recent years indicate that non-neutralizing Abs (nnAbs) may also exert significant anti-HIV functions (Milligan et al., 2015, Barouch et al., 2015) facilitated through Fc-mediated interactions with FcRs expressed on innate cells in the mucosa (Alter and Moody, 2010, Li et al., 2014, Chung et al., 2015). For example, anti-V1V2 IgG Abs capable of mediating Ab-dependent cellular cytotoxicity (ADCC) were induced by the candidate HIV-1 vaccine regimen in the RV144 trial (Pollara et al., 2014), which at relatively high levels correlated with reduced infection risk (Haynes et al., 2012, Yates et al., 2014). These observations provided optimism that vaccine-induced non-neutralizing, type-specific Abs may indeed contribute substantially to vaccine-induced protection and could be a more achievable goal for vaccine designs (Haynes and Mcelrath, 2013, Barouch et al., 2015). Thus, defining the key roles and comparing the efficacy of bnAb *vs*. nnAb functions in relevant models has been a major focus of recent investigations.

A surprising finding in the RV144 immune correlates analysis was that monomeric anti-Env IgA in plasma may mitigate otherwise protective IgG responses (Haynes et al., 2012, Tomaras et al., 2013). Both IgA and IgG antibody isotypes are key mediators of defense against pathogen invasion. Although IgG is more abundant in the lower female genital tract and male foreskin epidermis, IgA is the main Ab isotype in most mucosal compartments (Lemos et al., 2016, Mestecky et al., 2009). The two human subclasses of IgA, IgA1 and IgA2, can each exist as dimeric (dIgA) and multimeric forms comprising two or more monomeric IgAs covalently linked by a J-chain; multimerization enhances IgA avidity and its aggregation potential (Stieh et al., 2014, Woof and Russell, 2011). Compared to IgA1, IgA2 is the more prevalent subclass in the female genital tract and colon (Woof and Russell, 2011), and its shorter hinge region and additional disulfide bonds create a more compact, rigid structure with enhanced resistance to degradation by proteases present in the mucosa. The polymeric IgA receptor (pIgR) provides unidirectional transport of dIgA to the apical epithelial surface, where it complexes with secretory component (SC), resulting in secretory IgA (sIgA). Moreover, the glycosylation of IgA SC facilitates the interaction with mucus (Woof and Russell, 2011). Transport and maintenance of IgG in the GI and GU tracts are regulated by the neonatal Fc receptor (FcRn) on epithelial cells, which binds IgG at low pH and releases it at neutral pH (Pyzik et al., 2015, Tzaban et al., 2009). In the lower female genital tract, the acidic pH of the lumen favors retention of IgG at this site. In the gut, FcRn enables transport of IgG to the lumen where it can bind to its cognate antigen and then return as immune complexes for presentation to dendritic cells (Pyzik et al., 2015, Tzaban et al., 2009). Thus, FcRn may paradoxically also enable transcytosis of infectious HIV-1 virions carried in tow by IgG (Gupta et al., 2013). In summary, differences in the structure, transport and Fc-mediated effector functions can influence the role of IgG and IgA Abs in mucosal HIV-1 infection.

Despite its importance in mucosal host defense, the role of IgA in mucosal protection against HIV-1 transmission is less defined than that of IgG. To address this, we generated a mAb panel of IgG1 and various IgA isoforms, with particular emphasis on the more stable IgA2 subclass; these include a bnAb directed against the HIV-1 gp120 CD4 binding site (CD4bs) neutralizing epitopes, and nnAbs directed at gp120 C1 and V2 epitopes and the gp41 immunodominant domain (Bonsignori et al., 2011, Wu et al., 2011, Liu et al., 2013, Liao et al., 2013, Bonsignori et al., 2012). Moreover, we have expressed the CD4bs bnAb, CH31, as IgG1, and as monomeric, dimeric and secretory IgA2 (Zhang et al., 2016). We similarly expressed the IgG1, monomeric and dimeric IgA2 isoforms of the non-neutralizing gp41-specific mAb, 7b2 (Zhang et al., 2016, Santra et al., 2015). Using these mAbs, the impact of various IgA forms on the functional capacity of neutralizing and non-neutralizing mAbs was investigated in various assays and models of HIV-1 transmission. This panel has been extensively characterized in a variety of *in vitro* assays supporting their potential to exert various antiviral functions *in vivo*, such as phagocytosis (Tay et al., 2016), virus capture (Liu et al., 2013, Liu et al., 2014), virus aggregation (Stieh et al., 2014, R. Shattock, personal communication), blocking of virus binding to galactosyl ceramide (GalCer) (Dennison et al., 2014), ADCC (Tomaras et al., 2013, Pollara et al., 2014, Bonsignori et al., 2012) and neutralization (Santra et al., 2015). Here, we have evaluated the protective capacity of these mAbs *ex vivo* in a human vaginal tissue explant model and *in vivo* in an NHP intrarectal model of HIV-1 infection to identify key Ab properties associated with early mucosal protection that may guide the development of effective prevention strategies.

## 2 Materials and Methods

### 2.1 Ethics

Indian-origin rhesus monkeys used in the immunization studies were housed and maintained in an Association for Assessment and Accreditation of Laboratory Animal Care-accredited institution in accordance with the principles of the National Institute of Health. All studies were carried out in strict accordance with the recommendations in the Guide for the Care and Use of Laboratory Animals of the National Institutes of Health in BIOQUAL (Rockville, MD). BIOQUAL is fully accredited by AAALAC and through OLAW, Assurance Number A-3086. The animal protocol used in this study was approved by the BIOQUAL IACUC (#14-B080). All physical procedures associated with this work were done under anesthesia to minimize pain and distress in accordance with the recommendations of the Weatherall report, “The use of non-human primates in research”. Teklad 5038 Primate Diet was provided once daily by animal size and weight. The diet was supplemented with fresh fruit and vegetables. Fresh water was given *ad libitum*.

Human vaginal tissue, that would otherwise have been discarded, was obtained from healthy women (free of known malignancy) undergoing vaginal repair surgeries in the Department of Obstetrics and Gynecology at the University of Washington. These surgical remnants were collected without identifying patient information under a waiver of consent approved by the IRBs of the University of Washington and the Fred Hutchinson Cancer Research Center (IRB #1167).

### 2.2 Phenotypic Characterization of Vaginal Leukocytes

Vaginal tissues were maintained in ice cold saline solution or 1 × DPBS during transport and dissection. Damaged, inflamed or otherwise visibly irregular tissue was excised. To characterize leukocytes found both within and beneath the vaginal epithelium (*i*.*e*., whole vaginal tissue), the stroma was trimmed to leave approximately 2–3 mm thick tissues that were subsequently dissected into about 1 × 1 mm pieces and stored overnight in RPMI at 4 °C. The next morning, cells were isolated using a collagenase digestion protocol (McKinnon et al., 2014). For some experiments, intraepithelial leukocytes were specifically isolated in parallel. In these experiments, the vaginal stroma was trimmed to yield tissue approximately 4 mm thick, dissected into ~ 3 × 5 mm pieces, placed in cold DPBS containing 5 mM EDTA, and incubated at 4 °C under agitation. The next day the epithelium was peeled from the stroma as previously described (Hladik et al., 2007) and dissected into 1 × 1 mm pieces for collagenase digestion. Cells were suspended in DPBS and transferred to 96-well round bottom plates and stained with LIVE/DEAD fixable Aqua Dead Cell Stain kit (Life Technologies, Grand Island, NY, USA) in DPBS. Cells were washed with DPBS and then stained with various antibody panels for phenotyping (Table S1) in FACS wash (DPBS containing 2% FBS and 2 mM EDTA) for 30 min on ice. The cells were washed with FACS wash and fixed with 1% paraformaldehyde. Compensation controls were prepared using CompBead (BD) for Abs and ArC Amine Reactive Compensation Beads (Life Technologies) for the viability stain. Samples were acquired on LSRII flow cytometers (BD), equipped with 405 nm, 488 nm, 535 nm and 635 nm lasers. Fluorescence parameter PMTs were normalized using Rainbow Calibration Particles, Peak 7 (Spherotech, Lake Forest, IL, USA).

### 2.3 Monoclonal Antibodies Tested in *Ex vivo* and *In vivo* Mucosal Challenge Models

7B2 mIgA2, 7B2 dIgA2, CH29 mIgA, CH31 mIgA2, CH31 dIgA2, CH38 mIgA2, CH31 IgG and control CH65 mIgA were produced as described (Zhang et al., 2016). HG130 mIgA1, HG129 mIgA1 Abs were isolated from memory B cell cultures from RV144 vaccinees (Bonsignori et al., 2012). 7B2 IgG1 (AAA), CH38 IgG1 (AAA), CH54 IgG1 (4A), CH57 IgG1 (4A), CH58 IgG1 (4A), CH29 IgG1 (4A), CH90 IgG1 (AAA) were produced, optimized for FcRγIIIa binding as described (Shields et al., 2001) and used as described (Bonsignori et al., 2012, Pollara et al., 2014, Ferrari et al., 2011). Antibody b12 IgG was a gift from Dennis Burton, La Jolla, CA. Control human mAb CH65 is an influenza nAb (Whittle et al., 2011) and was produced as CH65 IgG1 AAA or 4A that are optimized for FcγRIIIa binding (Shields et al., 2001) or was produced as monomeric IgA2 (Zhang et al., 2016).

### 2.4 Viruses for *Ex vivo* Challenge

Previously described, replication competent, HIV-1 Env-chimeric reporter virus IMCs were redesigned to express the secreted nanoluciferase (NanoLuc™) reporter *in lieu* of the *Renilla* luciferase reporter (Edmonds et al., 2010). These viruses express heterologous Env ectodomains in an isogenic background (NL4-3) and upon replication, the nanoluciferase reporter is secreted into the culture supernatant facilitating kinetic monitoring of infection. Infectious HIV-1 snLuc reporter IMCs expressing the Env ectodomains of strains JR-CSF and Bal26 (referred to as snLuc.HIV-1_JR-CSF_ and snLuc.HIV-1_Bal26_, respectively) were generated for this study. An Env-defective construct, referred to as snLuc.HIV-1mssD (delta env virus), was also generated as a negative infection control. See Supplemental Methods for details regarding cloning, virus production and titering.

### 2.5 *Ex vivo* Vaginal HIV-1 Infection Assay

Vaginal tissues were collected and trimmed as described above to yield 2–3 mm thick strips of tissue, which were then cut into ~ 3 mm explants. Explants were washed thoroughly with cold DPBS (Gibco) and transferred (3 per well) into 48-well tissue culture plates (Costar). The explants were activated overnight at 37 °C with 0.6 μg/mL phytohemagglutinin (Remel) in maintenance media [RPMI (Gibco) supplemented with 10% heat-inactivated human serum AB (Gemini Bio-Products), l-glutamine, penicillin streptomycin, 500 U/mL of IL-2, 25 ng/mL of IL-7 and 5 ng/mL of IL-15 (Peprotech)]. In some experiments, fetal bovine serum (Gemini Bio-Products) was used instead of human serum. The next day, explants were challenged overnight (18–24 h) with replication-competent, HIV-1 Env-chimeric snLuc reporter viruses (5 × 10^5^–2 × 10^6^ IU, depending on virus), which were pre-incubated with mAbs of interest for 1 h. The challenge inoculum was collected the next day, and explants were washed thoroughly (5 times with DPBS and once with culture media) in the culture wells, taking care to minimize the loss of migrated cells from the explant cultures. Explants were cultured in maintenance media containing the mAbs of interest for 24–48 h. Subsequently, ~ 80% of the culture media was collected and replaced (without mAbs of interest) every 2–3 days for up to 21 days. In experiments where AZT or IDV (obtained from the NIH AIDS Reagent Program, Division of AIDS, NIAID), were used as positive controls of inhibition, they were pre-incubated with explants for 1 h prior to virus challenge and maintained in culture up through day 7 post-infection. In experiments where snLuc.mssD (delta env, Env defective reporter virus) was used as a negative control for infection, the input RLU was matched to that of the replication competent IMC used in the experiment. Inhibition was verified in at least four independent experiments, each utilizing tissues from a different HIV-1 susceptible donor, to account for the baseline infectivity rate (~ 80–85%) of cervicovaginal tissues challenged *ex vivo* (unpublished data; Astronomo et al. and (Dezzutti et al., 2013)).

### 2.6 Measuring HIV-1 Infection in Vaginal Explants *via* Luciferase Assay

Productive infection of vaginal explants was monitored by measuring snLuc activity in the culture media collected over the course of 21 days. The Nano-Glo Luciferase Assay System (Promega) was used with 20 μL of culture supernatants, measured in singlicate, according to the according to the manufacturer's instructions. Luminescence was measured on an MLX 96 Well Plate Luminometer (Dynex Technologies) and reported in relative light units (RLU). Variation in technical replicates, even performed on different days, was found to be insignificant. One-tailed Spearman's correlation analysis of reported *in vitro* neutralization potencies and observed *ex vivo* protection was done in GraphPad Prism.

### 2.7 Nonhuman Primate Studies

Mamu-A*01-negative, adult Indian-origin rhesus macaques weighing 3–6 kg were housed at Bioqual, Inc., Rockville, MD. Monkeys were randomly selected for sex. All monkeys were maintained in accordance with the Guide for the Care and Use of Laboratory Animals. All animal protocols were approved by the Institutional Care and Use Committee.

### 2.8 Pharmacokinetic Studies in NHP

*In vivo* pharmacokinetics (PK) studies were performed prior to passive protection studies for all Abs to determine the concentrations and the half-lives of the Abs in circulation and at the mucosal sites. Antibody administration in PK studies was performed by either of the two methods – i) systemic administration by IV route and ii) local instillation at mucosal site. In all PK studies where Abs were administered systemically, three rhesus monkeys were used per antibody tested. Abs were systemically infused at 50 mg/kg dose at 0 and 48 h. In PK studies where Abs were instilled at the mucosal sites, two rhesus monkeys were used per antibody and 5 mg antibody was instilled at 0 h in the rectal space in 1–2 mL volume. In all PK studies blood and rectal wecks were collected at regular intervals up to 5 days post-1st administration to determine the peak levels and half-lives of infused or instilled Abs in plasma and rectal space. Measurements of mAb levels in plasma and weck samples were done as previously described (Santra et al., 2015).

### 2.9 Passive Protection Studies in NHP With SHIV Challenge

In passive protection studies, two groups of rhesus monkeys (*n* = 6 or *n* = 8 per group) received two IV infusions with either the experimental antibody or the isotype-matched anti-influenza CH65 mAb at 50 mg/kg at 0 and 48 h. Six or eight animals per group were selected in order to determine statistical differences if all but one animal in one group showed a difference in outcome than in the other group using a two-tailed exact Wilcoxon test. For protection studies where monomeric (mIgA), dimeric (dIgA) and secretory (sIgA) IgA Abs were instilled at the rectal space, six monkeys were used per form of IgA mAb used. For these experiments one control group of six monkeys was used that received monomeric CH65 IgA. In each protection study, monkeys were challenged intrarectally with SHIV at the time when peak level of antibody was detected in rectal space as determined by the respective PK studies. Monkeys in CH38 mIgA2 and CH54 IgG1 passive protection studies were challenged with undiluted SHIV_Bal_ (~ 2 × 10^5^ TCID_50_) by the intrarectal route. In other studies monkeys were challenged intrarectally with 1:75 dilution of SHIV_SF162P3_, harvest 4 (ABL, Kensington, MD). Blood samples were collected at pre-infusion, pre-challenge and then weekly up to day 70 post-challenge. Plasma viral RNA levels were measured at all collected timepoints to determine protection from viral challenge (Santra et al., 2015). Briefly, viral RNA was extracted from plasma samples to make cDNA. The cDNA was used in qPCR reactions, which were run in duplicate for each sample.

### 2.10 Enumeration of Transmitted/Founder Viral Genomes Following High Dose SHIV_Bal_ Challenge

T/F genomes of viruses sequenced from rhesus macaque plasma post-infection were sequenced and enumerated as previously described (Santra et al., 2015). Comparisons of variant numbers between the treatment and control groups were analyzed by the two-tailed Mann-Whitney Wilcoxon rank sum test.

### 2.11 Dimeric IgA2 Binding to Rhesus Secretory Component

Binding of human and rhesus SC protein to dimeric IgA2 were determined in ELISA as described (Zhang et al., 2016). CH31 dIgA2 or 7B2 dIgA2 was first coated at the saturated amounts (2 μg/mL, 30 ng/well) in 384-well ELISA plates overnight at 4 °C, and then incubated with either human or rhesus SC protein at concentrations ranging from 50 μg to 0.023 μg/mL 4 °C followed by incubation with saturated amounts (2 μg/mL) of anti-SC MAb 9. After incubation, these plates were washed twice with PBS and then incubated with secondary antibody of horseradish peroxidase (HRP) conjugated goat anti-mouse IgG. These plates were then washed 4 times with PBS and developed with 30 μL/well TMB substrate (SureBlue Reserve, Gaithersburg, MD). The HRP reaction was stopped with 30 μL/well 1 M HCl and optical density (OD) of the reaction was measured at 450 nm.

### 2.12 Purification and Analysis of Antibodies

IgA mAbs were purified by peptide M agarose and size exclusion chromatography as described (Tomaras et al., 2013, Liao et al., 2004, Zhang et al., 2016). IgG Abs were purified by protein G columns as described (Zhang et al., 2016). Abs were characterized by SDS PAGE or blue native page and western blot analysis as described (Zhang et al., 2016).

### 2.13 ADCC Testing for the Selection of Macaques for CH54 IgG1 Passive Infusions

To ensure that the NK cells of all macaques chosen to receive the human CH54 IgG1 mAb had an appropriate FcR allele that could bind the CH54 Fc and could mediate ADCC against HIV-1_Bal_ IMC-infected target cells (Pollara et al., 2014), a series of macaque peripheral blood NK cells were assayed for this ability. Macaques were chosen based on NK cell ability to mediate CH54 ADCC of HIV-1_Bal_ IMC-infected targets. Thus, by using only macaques with this capability, if the CH54 IgG1 mAb did not protect against SHIV challenge, we could rule out an inability to bind the human CH54 Fc as the reason for lack of protection. The cryopreserved human CAVD002A-7 PBMCs with the FcγRIIIa phenotype F/F were obtained from the CT-VIMC repository as part of the Collaboration for AIDS Vaccine Discovery. The C1/C2 A32 IgG1 (AAA) mAb was provided by Dr. Haynes.

### 2.14 Neutralization Assays

Neutralization assays were performed in the TZM-bl assay as described (Li et al., 2005). Viruses for SHIV_Bal-P4_ and SHIV_SF162P3_ were grown in human PBMC.

## 3 Results

### 3.1 Leukocytes are in Vaginal Tissue to Engage in IgG and IgA Fc-Mediated Antibody Effector Functions

Several Ab functions may contribute to mucosal protection against vaginal HIV-1 infection, many of which are dependent on effector cell engagement *via* appropriate Fc receptors (FcRs). However, we have limited knowledge about FcR expression in human vaginal tissue. To understand whether FcR-expressing leukocytes are present to engage anti-Env mAbs in the human vaginal tissues in our model, we examined freshly isolated human leukocyte populations and their FcR expression patterns from vaginal tissue, derived either from vaginal epithelial sheets or whole mucosal tissue (including stroma and epithelium).

**Fig. 1:**
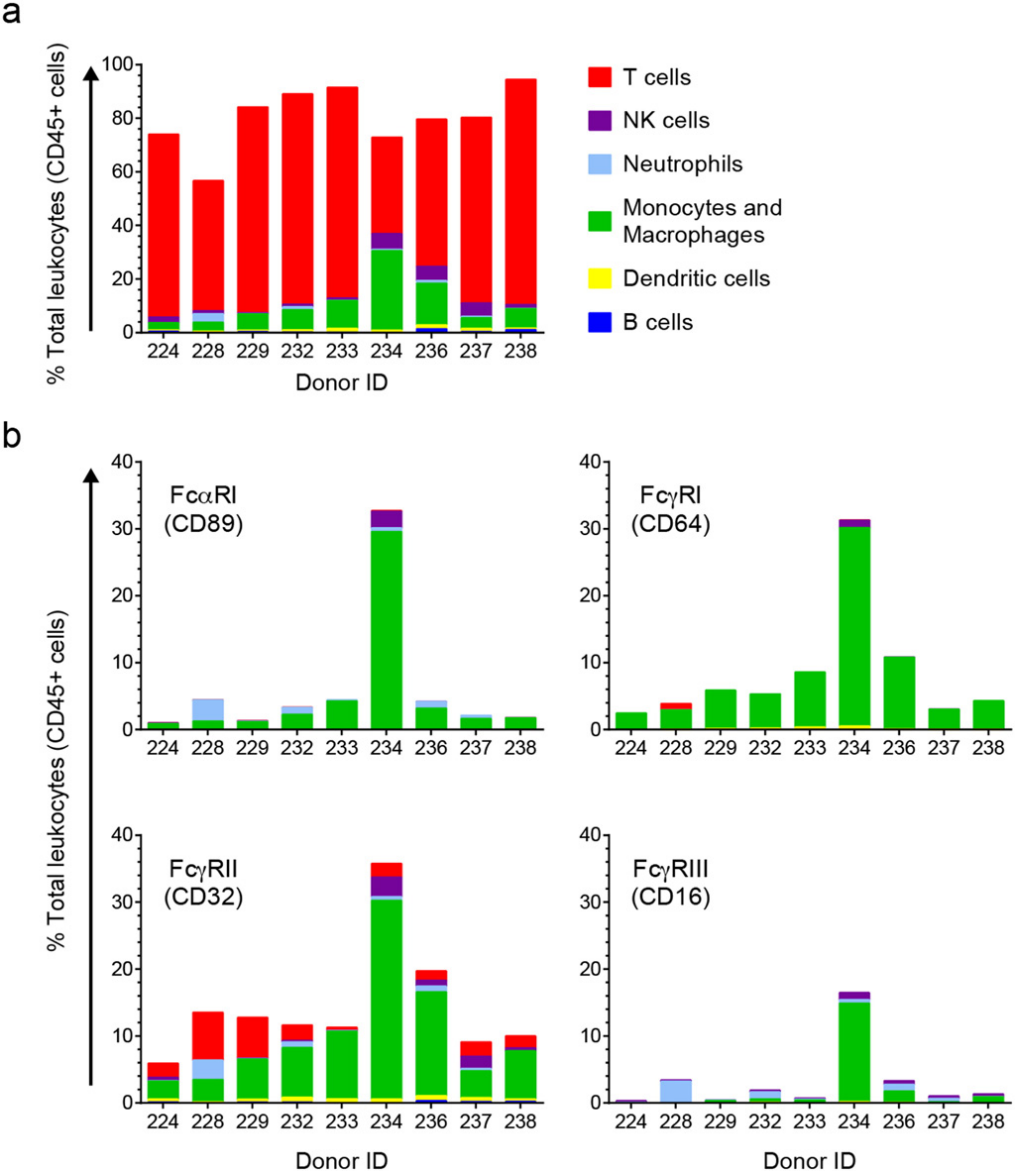
In human vaginal tissue, macrophages and monocytes are the main FcR-expressing effector cells and FcγRII is the most widely expressed FcR on vaginal leukocytes. Major leukocyte populations were identified in single cell suspensions isolated from whole vaginal tissue and their FcR expression profiles were determined by flow cytometry. (a) The frequency of each leukocyte population (*e*.*g*., B cells) identified in vaginal cell suspensions relative to total live leukocytes (CD45 +). (b) The proportion of vaginal leukocytes that express FcγRI, FcγRII, FcγRII or FcαRI. The contribution from each leukocyte population is show as a percentage of total live leukocytes.

The relative distribution of leukocytes (B cells, dendritic cells [DCs], macrophages and monocytes, neutrophils, natural killer [NK] cells and T cells) in human vaginal tissue of nine donors is shown in Fig. 1a (see Fig. S1 for the flow cytometry gating strategy and Table S1 for the flow cytometric fluorescent antibody panel). The FcR expression profiles for these populations are shown in Fig. 1b. While CD3 + T cells were more frequently identified than other leukocytes (Fig. 1a), macrophages and monocytes were the most abundant vaginal leukocyte population expressing FcRs and thus available to mediate both IgG and IgA effector functions (Fig. 1b). They were primarily observed in leukocytes isolated from whole tissue rather than epithelial sheets (representative example, Fig. S2). DCs were found in low abundance in both compartments; most intraepithelial DCs were Langerhans cells (HLA-DR +, CD1a +) (Figs. 1a and S2). In these analyses, CD3-CD56 + NK cell frequency and distribution within vaginal tissue was generally low but variable among donors (Fig. 1a). These findings are generally consistent with recent reports (Sips et al., 2016, Cheeseman et al., 2016a).

Macrophages, monocytes and neutrophils account for the majority of FcαRI + (CD89) leukocytes, though the total proportion of FcαRI + leukocytes was less than 6% in all but one donor (ID #234), whose leukocyte population frequencies and FcR expression profiles significantly deviate from the others. FcγRII (CD32) was identified on the greatest proportion of leukocytes (8–36%, median: 12%), including all macrophages and monocytes and the majority of neutrophils (Fig. 1b). FcγRI (CD64) was primarily expressed on monocytes and macrophages. Subsets of FcγRIII (CD16)-expressing leukocytes were primarily NK cells and neutrophils, both of which were rare in vaginal tissues, typically less than 1% of total CD45 + cells (Figs. 1a and S2). Of note, neutrophils express FcγRIIIb, which is functionally and structurally different from FcγRIIIa (Ravetch and Perussia, 1989, Selvaraj et al., 1988). These data suggest that macrophages and monocytes are the predominant FcγR- and FcαR-expressing effector cells accessible in human vaginal tissue, though they localize primarily below the epithelium. FcR-expressing neutrophils and NK cells, in general, were variable among donor tissues and typically in lower abundance.

### 3.2 Only mAbs With Broadly Neutralizing Activity Block HIV-1_JR-CSF_ in an *Ex vivo* Vaginal Infection Model

**Fig. 2:**
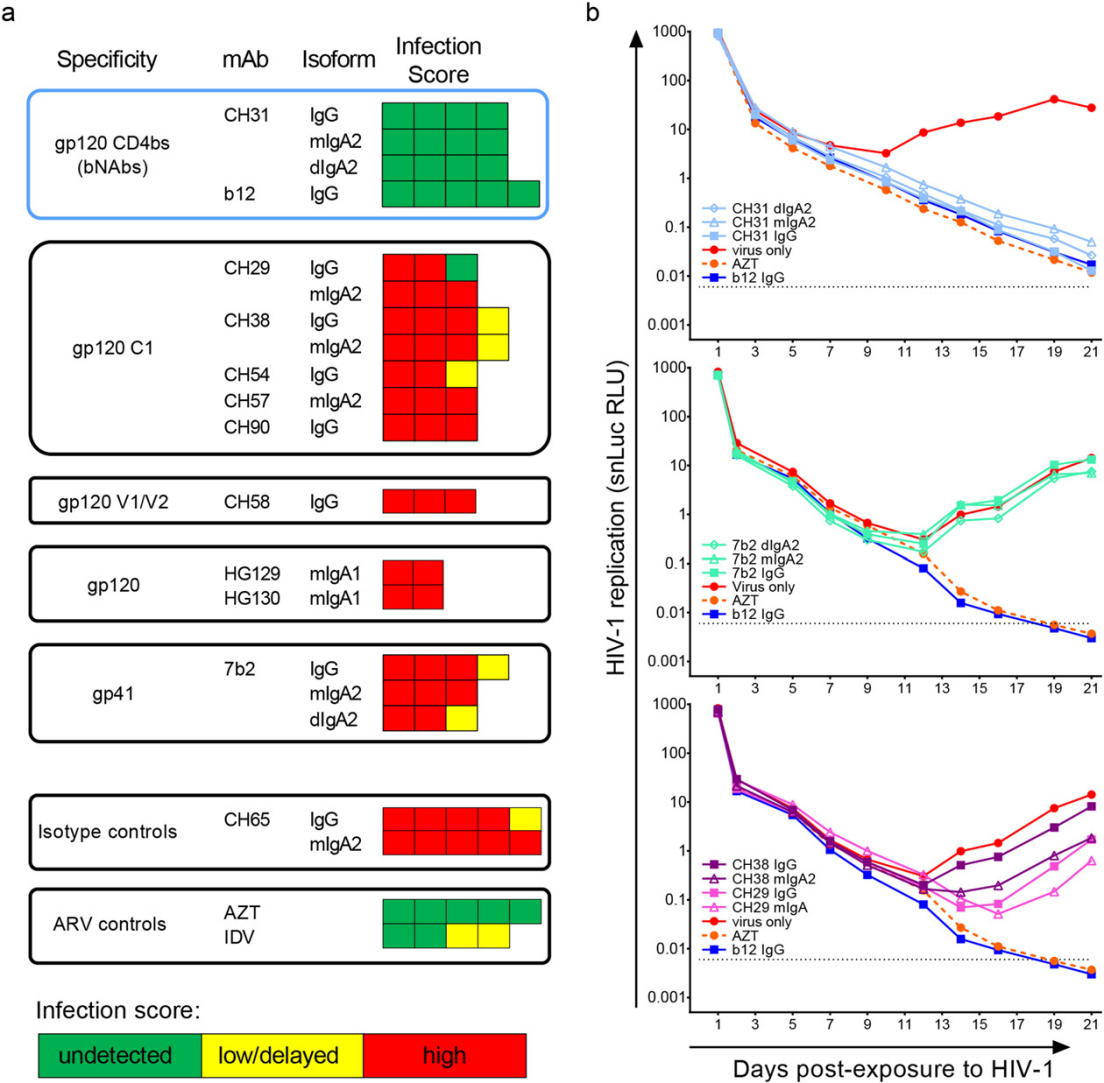
Broadly-neutralizing but not the non-neutralizing mAbs block snLuc.HIV-1_JR-CSF_ infection in human vaginal explants. (a) Summary of *ex vivo* HIV-1 inhibition results from kinetic snLuc infection assay conducted with tissue from 5 donors. Each square represents the result from an individual donor tissue for the listed mAb or small molecule. bnAbs are outlined in blue. (b) Representative data from vaginal explant infectivity model. PHA-activated vaginal tissue explants (3 per well) were exposed to snLuc-.HIV-1_JR-CSF_ (1 × 10^6^ IU/well) that was pre-opsonized with the indicated mAbs at 20 μg/mL. The next day, the inoculum was collected (Day 1 samples in plots above), the tissues were washed several times and individual mAbs were added back at 20 μg/mL to the culture media. Explants were maintained in culture for up to 21 days, with 80% media collection and replacement every 1–3 days. Decay in snLuc activity (RLU) after day 2 indicates inhibition of HIV-1 infection. By contrast, an increase or low level maintenance in snLuc activity over time indicates productive infection. Delayed infection kinetics were defined as productive infection that is delayed by at least two time points compared to the matched virus only conditions. The CH65 IgG and IgA mAbs were included as isotype controls. Indinivir (IDV, protease inhibitor), Zidovudine (AZT, reverse transcription inhibitor), and b12 IgG were included as positive controls for virus inhibition. The antiretrovirals (ARV) were maintained up to day 7 post-exposure.

We developed a human, whole vaginal tissue explant model of *ex vivo* HIV-1 infection to assess antibody-mediated protection against acute infection, using a panel of HIV-1 Env-specific bnAbs and non-neutralizing mAbs (Fig. 2a), in the presence of tissue resident effector and target cells described above. The mAbs were tested at 20 μg/mL, a dose achievable through active or passive immunization. A secreted NanoLuc™ (snLuc)-expressing, replication competent, infectious molecular clone (IMC), snLuc.HIV-1_JR-CSF_, was used as the challenge virus. In this assay, inhibition of viral replication is shown by the progressive decline in snLuc reporter activity in the culture supernatant over time, which reflects the dilution of the residual snLuc in the inoculum. The reverse transcriptase inhibitor zidovudine (AZT), the protease inhibitor indinivir (IDV) and the well-characterized CD4bs bnAb b12 IgG (Hu et al., 2004) were used as positive inhibition controls in this assay. The influenza-specific mAbs CH65 IgG and mIgA2 (Whittle et al., 2011) were included as isotype-specific negative controls (summarized in Fig. 2a).

The Env-specific mAb panel represented various Ab specificities, isotypes and functions (Fig. 2a). These included IgG and IgA isotype variants of a VRC01-like bnAb (CH31) and several nnAbs exhibiting known alternative effector functions such as ADCC (gp120 C1-specific mAbs), phagocytosis (V2-specific CH58 and gp41-specific 7b2), virus capture (7b2), and GalCer blocking (CH38 IgA2). Many of these mAbs displayed multiple *in vitro* functions and the isotype variants of the same mAb have altered functional profiles (Bonsignori et al., 2012, Dennison et al., 2014, Liu et al., 2013, Pollara et al., 2014, Santra et al., 2015, Tomaras et al., 2013, Tay et al., 2016, Zhang et al., 2016). *In vitro* ADCC assay parameters influence the potency ascribed to gp120 C1-specific mAbs (Bonsignori et al., 2012, Veillette et al., 2014, Von Bredow et al., 2016) and the accessibility of these CD4i epitopes are not well-understood *in vivo*, warranting investigation of these mAbs in more physiologically relevant models. The nnAbs that mediate effector functions such as virus capture and phagocytosis may not be able to completely impede infection but could limit infectivity if sufficient time for opsonizing the infectious virus particles were provided (Tay et al., 2016). Therefore, to enhance detection of antiviral activity, the mAbs were allowed to bind HIV-1 virions first before the vaginal tissue challenge. Furthermore, to model events during passive or active immunization, mAbs were added back once into the culture immediately after washing off the inoculum.

As expected, both AZT (maintained for 7 days in explant culture) and b12 IgG completely inhibited *ex vivo* infection in vaginal tissue (Fig. 2a and B). IDV-mediated suppression of infectious virion production and spread was apparent over the seven days during which the drug was maintained in culture, eventually resulting in either a low-level or non-productive infection (Fig. 2a). Conversely, the CH65 isotype controls had no effect on HIV-1 infectivity (Fig. 2a and b).

Similar to b12 IgG, the CD4bs bnAbs CH31 IgG, mIgA2 and dIgA2 also consistently blocked snLuc.HIV-1_JR-CSF_ infection to undetectable levels. (Fig. 2a and b, upper panel). By contrast, the nnAbs (7b2, CH38, CH29, CH54, CH57, CH90, CH58, HG129 and HG130) and their various isoforms did not inhibit this virus in the *ex vivo* model (Fig. 2a and b, middle and lower panels). Notably, the IgG nnAbs were modified to enhance binding to FcγRIIIa (Shields et al., 2001). These data indicated that CD4bs bnAbs provided superior protection against early events of HIV-1 infection with free virus in vaginal tissue, compared to mAbs that lacked bnAb activities but had the capacity to mediate antiviral functions such as ADCC and virus capture. The sporadic observations of low or delayed infectivity (Fig. 2a) more likely reflected inherent variation in tissue explant infectivity that has been previously reported (Dezzutti et al., 2013, Merbah et al., 2012). Thus, nnAbs may be less efficient in preventing acute HIV-1 infection, especially those most reliant on ADCC activity, and require additional time or the influx of additional effector cells (*e*.*g*., NK cells and neutrophils) to curb HIV-1 infection.

### 3.3 Non-neutralizing IgG and mIgA2 Abs do not Protect Macaques From Intrarectal SHIV_Bal_ Challenge

**Fig. 3:**
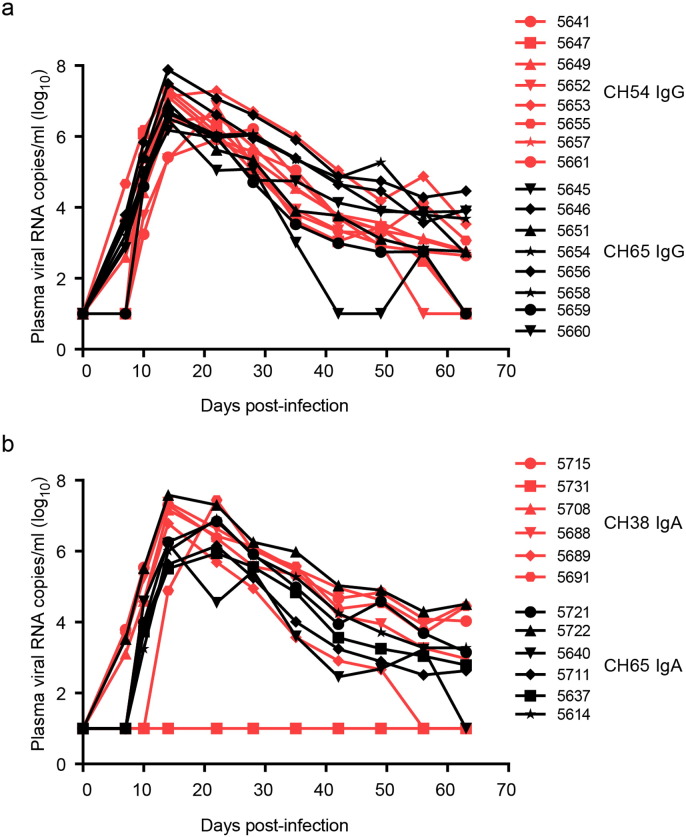
Passive infusion of CH54 IgG or CH38 mIgA2 does not protect rhesus macaques against high dose mucosal SHIV challenge. Groups of Indian-origin rhesus macaques received two IV infusions of either CH54 IgG (a), CH38 mIgA2 (b) or isotype matched anti-flu mAb CH65 at 50 mg/kg. Six hours post-1st infusion monkeys were challenged with SHIV_Bal_. Plasma viral RNA levels of individual monkey are shown.

In parallel studies, we investigated the protective efficacies of gp120 C1-specific non-neutralizing mAbs, CH54 IgG and CH38 mIgA2, against rectal transmission using a NHP high-dose intrarectal SHIV_Bal_ challenge model. Indian-origin rhesus macaques were pre-screened for FcR alleles that could bind to the human Fc of CH54 IgG1 using an ADCC assay. Only those animals with NK cells demonstrating > 20% specific killing were included in the study cohort (Fig. S3). The treatment group (*n* = 8 for CH54 IgG; *n* = 6 for CH38 IgA2) received intravenous (IV) infusions of mAb at 0 and 48 h at a dose of 50 mg/kg per infusion, while the control groups received the matched isotype control CH65 mAb IgG (*n* = 8) or IgA2 (*n* = 6) at the same time using the same dose. All animals were challenged at 6 h post first infusion, which corresponded to peak mucosal Ab levels (Fig. S4). Overall, neither CH54 IgG nor CH38 mIgA2 treatment inhibited infection by SHIV_Bal_ challenge, nor did they dampen the peak and post-peak viremia (Fig. 3). However, one CH38 IgA2-infused macaque (#5731) remained uninfected throughout the study, possibly due to commonly observed variation in the intrarectal challenge model rather than mAb-mediated protection as shown in the other five animals in the treatment group.

Although the non-neutralizing gp120 C1-specific mAbs were not protective *in vivo* in the NHP challenge model, we further examined if these mAbs had the capacity to restrict the number of transmitted/founder (T/F) virus variants. As shown in Table 1, a range of 25–43 sequences were used to estimate the numbers of T/F viruses. The median number of T/F variants was 3.5 in both the CH54 IgG mAb and the CH38 mIgA2 treatment groups; the median number of T/F variants was 5.5 in the CH65 IgG and 5.0 in the mIgA2 control groups (Table 1). These findings indicate that neither gp120 C1-specific mAb significantly limited the number of T/F virus variants in comparison to the isotype control mAb groups (*P* = 0.59 and *P* = 0.9, respectively). Thus, even at peak mAb levels (1.5–9.7 μg/mL CH38 mIgA2 or 5–22 μg/mL CH54 IgG) in rectal secretions (Fig. S3), neither CH38 mIgA2, which blocks gp120 binding to the alternative receptor GalCer on epithelial cells (Dennison et al., 2014), nor CH54 IgG, which mediates ADCC *in vitro* (Bonsignori et al., 2012, Pollara et al., 2014) could protect against SHIV_Bal_ acquisition or dampen viral replication after high-dose intrarectal challenge. These results may reflect insufficient accessibility of the CD4i epitopes (Veillette et al., 2014, Von Bredow et al., 2016).

### 3.4 A combination of Five IgG nnAbs Does not Inhibit *Ex vivo* HIV-1 Vaginal Infection

**Fig. 4:**
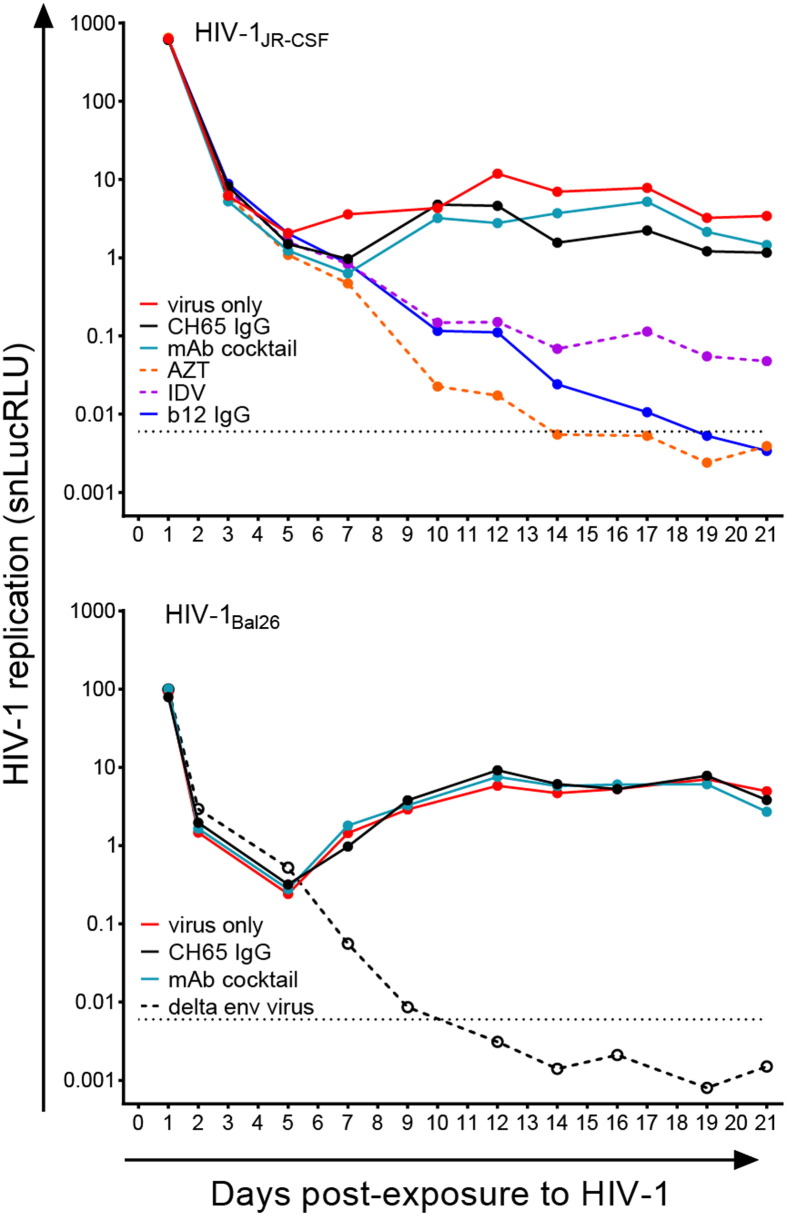
HIV-1-specific, non-neutralizing IgG mAbs in combination do not inhibit *ex vivo* HIV-1 infection of human vaginal explants. Representative data from two separate experiments in two donor tissues showing infection with snLuc.HIV-1_JR-CSF_ and (top) snLuc.HIV-1_Bal26_ (bottom). Three IgG mAbs directed against the C1 region of g120 (CH29, CH54 and CH57), one against the gp41 immunodominant (7b2 IgG) and one against a V1/V2 non-glycan conformational epitope (CH58 IgG) were combined at 4 μg/mL each, for a total of 20 μg/mL anti-HIV-1 mAb cocktail. These experiments were conducted as described in Fig. 2 with the following modifications: Culture media was supplemented with 10% heat-inactivated human serum AB in lieu of FBS, and an env-deleted snLuc reporter construct (delta env) was included as a negative control for snLuc.HIV infection.

Our vaginal and intrarectal challenge models both indicate that used alone, functional anti-HIV-1 Env non-neutralizing IgG and IgA2 were not protective against mucosal infection. However, non-neutralizing Abs have been shown to exert synergistic activities *in vitro* when measured in combination (*e*.*g*., V2 and C1 Abs (Pollara et al., 2014)). We used the vaginal explant model to evaluate the hypothesis that the protective capacity of a mixture of functional, but non-neutralizing, mAbs targeting multiple HIV-1 epitopes could act synergistically to protect against HIV-1 exposure. In these experiments, equal concentrations of five IgG mAbs (CH29, CH54, CH57, CH58 and 7b2) with specificities against three different Env regions were combined for a total concentration of 20 μg/mL anti-HIV-1 IgG. These, and subsequent explant experiments, were conducted in the presence of 10% human serum to potentially replicate the *in vivo* scenario where circulating IgG can influence FcR function; otherwise the experimental setup was similar to that used in the evaluation of individual mAbs. While a single bnAb, b12 IgG, at 20 μg/mL completely inhibited snLuc.HIV-1_JR-CSF_, the cocktail of five non-neutralizing anti-HIV mAbs showed no inhibitory effect (Fig. 4, upper panel). Moreover, the non-neutralizing mAb cocktail was unable to inhibit snLuc.HIV-1_Bal26_ infection (Fig. 4, bottom panel), which is relevant to the multiple *in vitro* functions these nnAbs demonstrated against the Bal01 or related Bal26 strains.

### 3.5 The bnAb CH31 IgG Shows Greater Protection Than the IgA2 Variants Against Intrarectal SHIV_SF162P3_

**Fig. 5:**
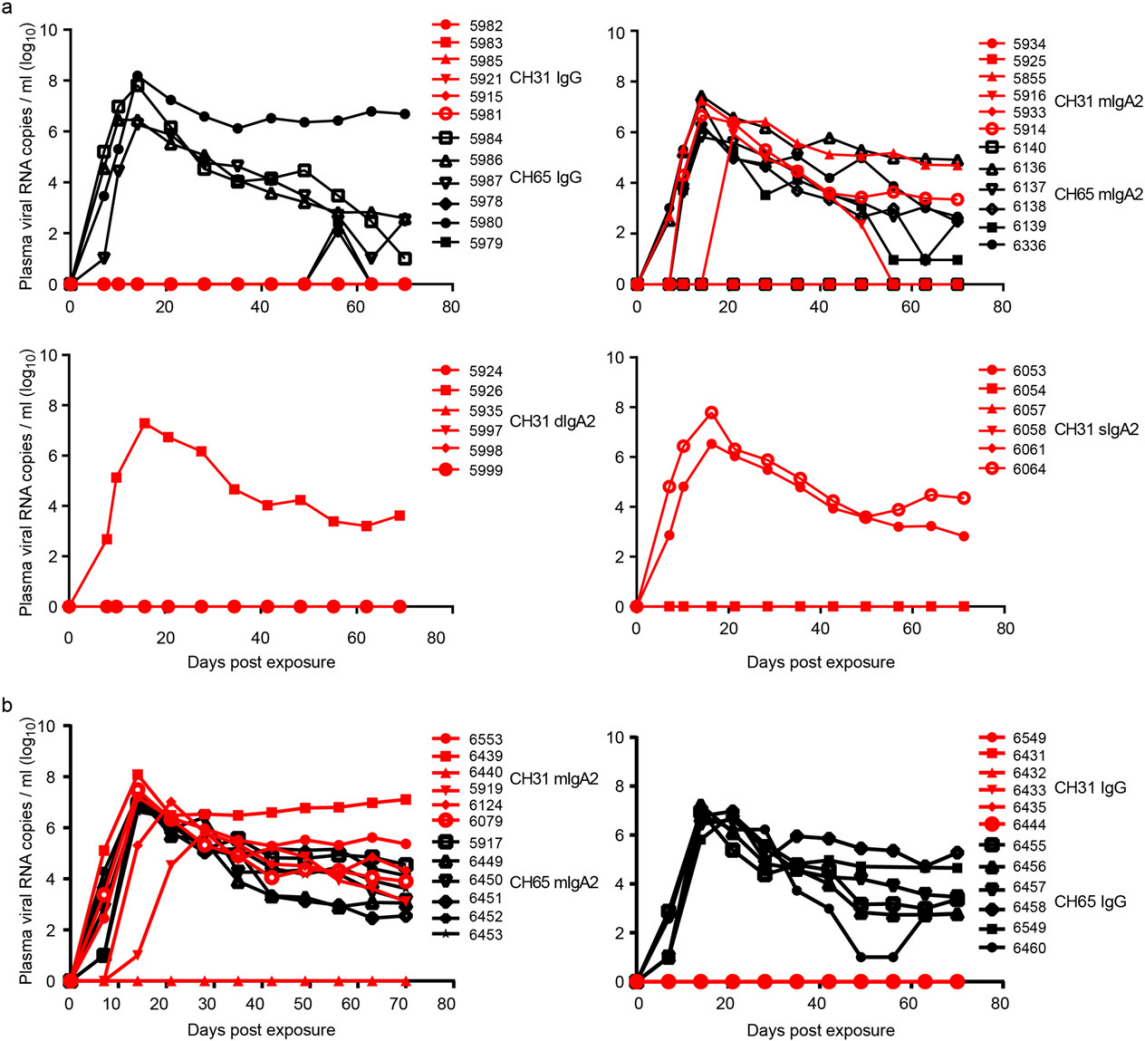
Protection by CH31 mAbs against SHIV_SF162P3_ intrarectal challenge. Plasma viral RNA levels following high dose rectal challenges with SHIV_SF162P3_ in monoclonal antibody-treated rhesus monkeys. (a) Rhesus monkeys were treated by rectal instillation with four different isotypes of CH31 monoclonal antibody including CH31 IgG, CH31 mIgA2, CH31 dIgA2 and CH31 sIgA2 followed by high dose intra-rectal viral challenge. Pavilizumab or CH65 IgG or CH65 mIgA2 were used as control. (b) Monkeys were passively infused with either CH31 IgG or CH31 mIgA2 prior to high dose rectal challenge. Pavilizumab or CH65 IgG or CH65 mIgA2 were used as control.

The protective capacity of CD4bs CH31 neutralizing mAbs against *in vivo* mucosal challenge was first evaluated by local instillation of the mAb in rectal mucosa followed by single, high-dose challenge with SHIV_SF162P3_. A total of 5 mg of either CH31 IgG, mIgA2, dIgA2 or sIgA2 mAb in 1–2 mL of PBS was instilled into the rectal mucosa of Indian-origin rhesus monkeys (*n* = 6 per CH31 bnAb isotype variant). Isotype-matched control mAbs, CH65 IgG and mIgA2, were similarly administered at the same dose. The homogeneity of all mAb preparations was verified by size exclusion chromatography and the binding of CH31 dIgA2 to rhesus and human secretory component was verified by ELISA (Zhang et al., 2016). Given the neutralization potency of these mAbs (Table S2), the more stringent SHIV_SF162P3_ intrarectal challenge was performed immediately after local installation. All animals infused with CH31 IgG were protected while those that received the control CH65 IgG became infected, though 2 animals had delayed viremia (Fig. 5a). Although the CH31 IgG mAb provided complete protection in all animals infused (6/6 animals), the various IgA mAb isoforms were only partially effective (Fig. 5a). Three out of six animals receiving CH31 mIgA2 were protected while all control animals receiving CH65 mIgA2 became infected; 5 of the 6 animals in the dIgA2 group and 4 out of 6 animals in the sIgA2 group were protected (Fig. 5a).

Pharmacokinetic (PK) measurements of the CH31 mAbs from the rectum following local instillation indicate large variations in mAb concentration depending on the isotype and the animals within the group. The highest peak mAb concentrations were measured for the IgG isotype (between 250 and 601 μg/mL) followed by the mIgA2 isotype (25.9–524 μg/mL). Much lower concentrations of dIgA2 (1.25–34.5 μg/mL) and sIgA2 (1.26–3.76 μg/mL) were detected at the rectal mucosa (Fig. S5A). Differential interactions with mucus have recently been observed for IgG and IgA in the female genital tract (Fahrbach et al., 2013), which may also apply to the gastrointestinal tract. Differences in how IgG and mIgA2, dIgA2 and sIgA2 isotypes interact with mucus and FcRs (*e*.*g*. FcRn and pIgR) may differentially impact the ability to measure IgG and IgA concentrations in the rectal secretions. Nonetheless, given the same dose of mAb applied and immediate challenge at the same locale, there was no apparent advantage in delivering IgA isotypes of the CD4bs bnAb CH31 intrarectally, despite the natural predominance of IgA isotypes in the GI tract.

The superiority of CH31 IgG compared to its mIgA2 counterpart was also apparent in NHP passive prophylaxis studies when administered intravenously. Two doses of mAbs at 50 mg/kg were administered at 0 and 48 h, followed by intrarectal challenge with SHIV_SF162P3_ at times corresponding to the peak mucosal mAb levels (1 h post-first infusion for the mIgA2 and 1 h post-second infusion for the IgG) as determined in previous PK studies (Fig. S5B). Significantly lower peak levels of mIgA2 (0.9–5.2 μg/mL, median = 1.9 μg/mL) compared to IgG (15.0–24.9 μg/mL, median = 16.7 μg/mL) were measured in rectal secretions. Not surprisingly, only one animal (1/6) that received the mIgA2 variant was protected compared to all the animals in the CH31 IgG group (Fig. 5b).

Isotype variants of the same mAb have been reported to have different affinities due to the influence of the constant heavy chain domains (Tudor et al., 2012). These domains can differentially influence the structure of the antigen-combining site and/or the overall flexibility of the Ab structure, both of which can change the affinity of the Ab for its epitope and consequently, its neutralization potency (Janda et al., 2016). To relate these *in vivo* findings to those *in vitro*, neutralization potency of the various CH31 mAbs was assessed using the TZM-bl assay and defined by IC50 and IC80 measurements. The IgG isotype is at least 10-fold more potent than any of the IgA2 variants (Table S2)·Although dIgA2 and sIgA2 CH31 variants were less effective at neutralization than mIgA2 (~ 2.5-fold and 4-fold higher IC50s, respectively), these dIgA2 variants captured more virions than mIgA2 and had greater potential to aggregate virus (R. Shattock, personal communication). Taken together, neutralization potency seems to be the predominant factor in the overall protective capacity of CH31 mAbs. While dimeric IgA2 variants may partially compensate for their inferior neutralization abilities by aggregating virions, the net effect falls short of their IgG counterpart.

### 3.6 *Ex vivo* Efficacy of bnAb Isotype Variants Parallel Their Relative *In vitro* Neutralization Potencies

**Fig. 6:**
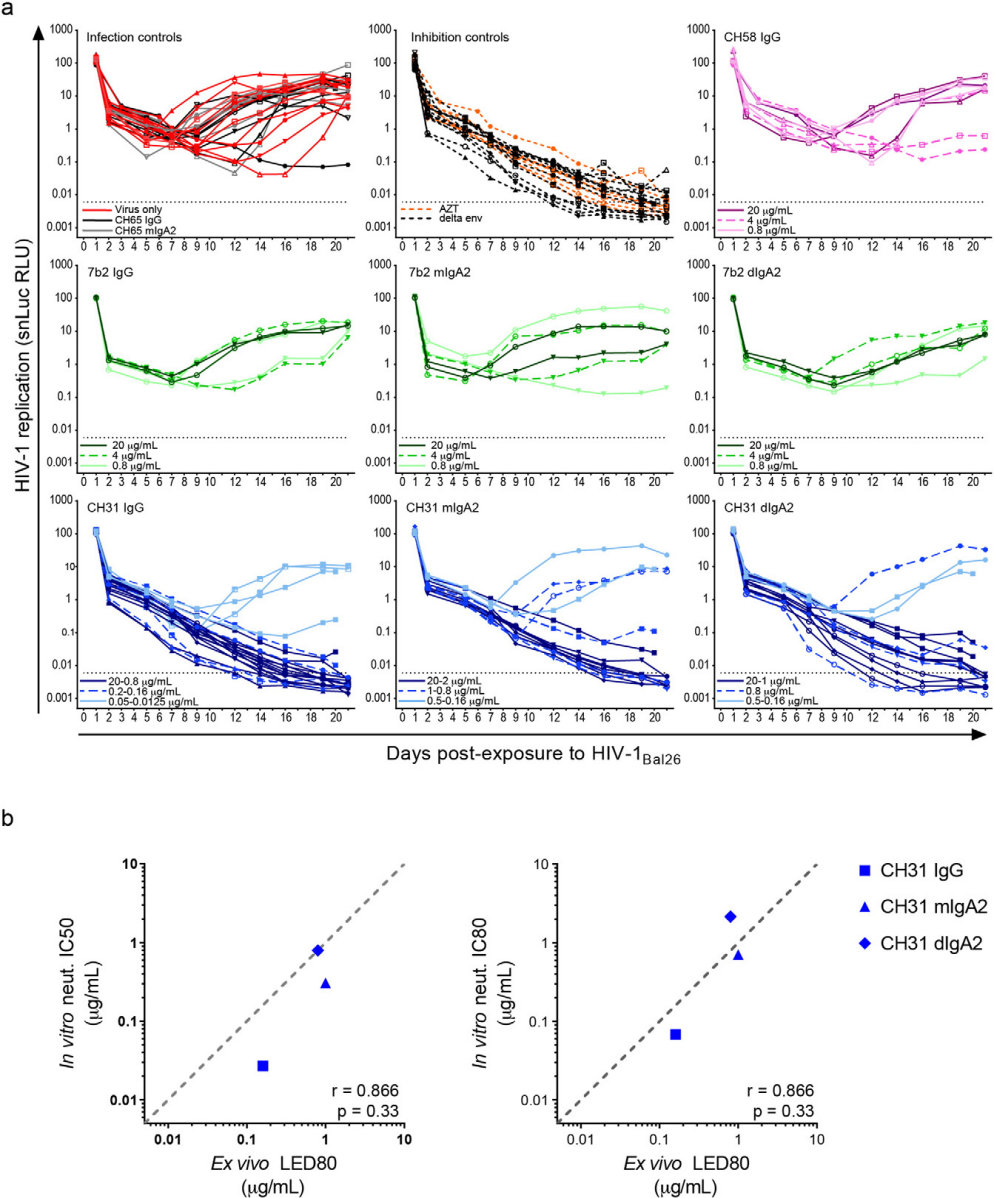
*Ex vivo* protection of human vaginal explants from HIV-1 infection by CH31 bnAbs reflects the relative potencies of each isotype variant in classic *in vitro* neutralization assays. (a) Summary of *ex vivo* snLuc.HIV-1_Bal26_ infection of vaginal explants in the presence of serially diluted isotype variants of 7b2 and CH31. Each line represents the readout from a single infection well containing 3 explants; symbols correspond to donors; and the 3 color gradients correspond to Ab concentrations that are considered high, medium and low. For 7b2 and CH58 mAbs these concentrations were 20, 4 and 0.8 μg/mL, respectively. For CH31 mAbs for which additional concentrations were tested, the individual infection curves were categorized into 3 color intensity groupings coinciding with the dose-response inhibition profiles of each isotype variant. Infection curves for (1) all mAb concentrations that consistently blocked infection to undetectable levels are depicted in dark blue; (2) the lowest concentration range that effectively blocked infection in most donor tissues are shown in medium blue; (3) and lower mAb concentrations that showed limited or no impact on HIV-1 infection are shown light blue. (b) The relationship between *ex vivo* (LED80) protection and *in vitro* neutralization potency IC50 (left) and IC80 (right) for CH31 mAbs. Neutralization IC50 and IC80 values were taken from (Table S2) except for the IC50 value for CH31 IgG which was taken from CATNAP (median of all reported values), www.hiv.lanl.gov. LED80 values were taken from Fig. S5. One-tailed Spearman's correlation analysis was done in GraphPad Prism.

The functionality of 7b2 and CH31 mAbs against HIV-1_Bal26_ strain was revisited since several *in vitro* functions for these mAbs have been demonstrated against this strain (or the highly related Bal01). Importantly, 7b2 mAbs capture infectious and non-infectious HIV-1_Bal_ and SHIV_Bal_ virions (Liu et al., 2014, Liu et al., 2013), mediate Ab-dependent monocyte phagocytosis of these virions (Tay et al., 2016, Santra et al., 2015). Although classified as a non-neutralizing mAb in standard PBMC and TZM-bl assays, 7b2 IgG was shown to mediate neutralization of HIV-1_Bal_ in blood monocytes and peritoneal macrophages (Santra et al., 2015). Moreover, 7b2 dIgA2 has the potential to aggregate HIV-1_Bal_ virions. 7b2 mAbs were tested at 0.8, 4 and 20 μg/mL against vaginal *ex vivo* snLuc.HIV-1_Bal26_ challenge to encompass the concentration range at which these Abs demonstrated antiviral functions *in vitro*, and to assess possible Ab prozone effects. Dose-dependent inhibition of snLuc.HIV-1_Bal26_ was not observed for any of the 7b2 mAbs in this explant model (Fig. 6a). Indeed, snLuc.HIV-1_Bal26_ infection in the presence of 7b2 mAbs showed similar infection kinetics as control infections conducted with virus alone, with isotype controls or with CH58 IgG recognizing a V1/V2 epitope not found on HIV-1_Bal26_ Env. These results suggest that in the absence of classic neutralizing activity, other antiviral Ab functions on their own do not significantly impede the early steps of HIV-1 infection in vaginal tissue.

By contrast, the same concentration range (0.8–20 μg/mL) of CH31 isotype variants strongly, if not completely, inhibited snLuc.HIV-1_Bal26_ infection of vaginal explants (Fig. 6a). At lower concentrations, breakthrough infection with CH31 mIgA2 and CH31 dIgA2 was observed. However, CH31 IgG consistently blocked snLuc.HIV-1_Bal26_ infection at concentrations as low as 0.16–0.2 μg/mL. To better quantify the *ex vivo* efficacy of mAbs in our explant model, the percent inhibition of overall virus replication (area under the curve, AUC), relative to matched donor infection controls, was determined (Fig. S6). By this metric CH31 mIgA2 reduced snLuc.HIV-1_Bal26_ infection by at least 78% in 4 of 5 donor tissues at 0.8–1 μg/mL while dIgA2 reduced infection by at least 80% in 5 out of 6 donor tissues at 0.8 μg/mL. At least 98% inhibition was achieved by CH31 IgG in 3 out of 3 donor tissues at 0.16–0.2 μg/mL and 80% inhibition was observed at 0.0125 μg/mL, though this concentration was only tested once.

To more formally assess the relationship between classic *in vitro* neutralization potency (as measured against pseudovirus in TZM-bl assays) and *ex vivo* efficacy, the lowest effective dose for 80% inhibition *ex vivo* (LED80, Fig. S6) was compared to the reported *in vitro* neutralization potencies (IC50 and IC80 values) for CH31 mAbs. While strong positive correlations were found for each comparison (Spearman *r* = 0.866), neither was statistically significant (*P* = 0.33) because of the small sample size (Fig. 6b). Nonetheless, Fig. 6b illustrates that differences in protective efficacy of CH31 isotype variants against mucosal HIV-1 infection clearly reflect differences in isotype-specific neutralization potencies against the matched strain, which was mirrored in our NHP challenge experiments. The comparison of LED80 to IC80 also shows that effective *ex vivo* protection can be achieved at concentrations closely corresponding to the neutralization IC80 values, suggesting that the protective capacity of these bnAbs in our vaginal infection model is largely explained by their ability to neutralize the challenge virus. Interestingly, CH31 dIgA2 protected vaginal tissue against HIV-1 infection better than it neutralizes virus *in vitro*, which likely reflects added gains in overall inhibition from its viral aggregation capabilities (Fig. 6b). Thus, the key findings from our *ex vivo* vaginal infection model are consistent with those from our intrarectal NHP challenge model.

## 4 Discussion

We sought to define properties of anti-Env Abs critical for mucosal HIV-1 protection, addressing two major issues in the vaccine field: the need to prioritize induction of IgA rather than IgG, and broadly-neutralizing activities rather than other antiviral functions. Given the dearth of female macaques and the extensive mAb panel available, we adapted our human vaginal explant model (Hladik et al., 2007, Mcelrath et al., 2010), containing HIV target and effector cells in their proper tissue architecture (Hladik et al., 2007, Mckinnon et al., 2014), to investigate Ab-mediated protection during the earliest events in vaginal infection, before seeding the viral reservoir. Only the bnAbs consistently protected against viral challenge; mAbs with known Fc-effector activities but lacking potent neutralizing function were not protective when tested alone or together. Of note, Cheeseman and colleagues found similar results to those reported here in *ex vivo* explants (Cheeseman et al., 2016b).

We developed a secreted NanoLuc™ reporter virus system building upon methods for creating replication-competent HIV-1 IMCs expressing the Renilla Luc reporter (Edmonds et al., 2010, Seay et al., 2015). This system has the advantage of monitoring HIV-1 infectivity kinetics using a simple, two-step Luc assay using different Envs in an isogenic virus background. Thus, this *ex vivo* explant assay format is well-suited to assess Ab-mediated inhibition and may be tailored to screen clinically-infused bnAbs and vaccine-induced polyclonal Abs with selected virus panels.

We determined relative tissue distributions of FcR-expressing leukocytes to define their accessibility in the *ex vivo* infectivity model to engage in Fc-binding Ab-mediated protection. We and others have demonstrated that CD4 + T cells are the first and predominant target cells for HIV-1 infection in the vaginal epithelium (Hladik et al., 2007), where few FcR-bearing effector cells other than DCs, particularly LCs, were found. Though well-positioned to engage Ab-bound virions or infected cells, the *in vivo* outcomes of such interactions are unclear due to the potential for HIV-1 to dysregulate DC function (Hladik and Mcelrath, 2008, Ahmed et al., 2015). By contrast, monocytes and macrophages expressing FcRs for IgG and IgA were relatively abundant, but were localized beneath the epithelium. Moreover, NK cells capable of mediating ADCC were rare throughout the tissue, in line with recent reports (Sips et al., 2016, Cheeseman et al., 2016a). Of note, DCs and monocytes and macrophages are also potential target cells and IgG nnAbs and nAbs have been shown *in vitro* to inhibit their infection by FcR-dependent (Holl et al., 2004, Holl et al., 2006a, Holl et al., 2006b, Peressin et al., 2011, Perez et al., 2009) and independent mechanisms (Lederle et al., 2014). Conceivably, these interactions could provide a second line of defense.

Importantly, the relative frequency and distribution of monocytes, macrophages and NK cells in vaginal tissue may limit their timely recruitment to efficiently engage with Abs to prevent initial CD4 + T cell infection in this model. This may partially explain why even a cocktail of IgG nnAbs was ineffective, despite previously described synergy and enhanced cooperativity in HIV-1 inhibition by nnAbs (Moog et al., 2014, Pollara et al., 2014). By contrast, bnAbs can exert immediate inhibition, the potency of which corresponds to their *in vitro* neutralization potencies against the challenge virus. The balance of inhibitory and activating FcγRII, the most prevalent FcR in these tissues, and innate viral responses can also influence the net impact of HIV-1 Abs on early infection. These factors warrant further investigation but were beyond the scope of this study.

We extended our investigations to a high-dose rectal challenge model as vaginal and rectal mucosae are anatomically and immunologically distinct compartments (Mcelrath et al., 2013, Mitchell et al., 2014, Cheeseman et al., 2016a, Sips et al., 2016), and the estimated risk of receptive anal intercourse is greater than that for receptive vaginal intercourse (Patel et al., 2014). Also, because neither the GalCer-blocking CH38 IgA2 nor ADCC-mediating CH54 IgG capture infectious virions, their greatest activity may be against cell-associated Env and better assessed in an *in vivo* challenge model. The mAb infusion schedules were designed to maintain mAb levels during the window that the first foci of infection would be established. Despite the potential for effector cell recruitment from the periphery, both nnAbs had no impact on SHIV acquisition, replication or on the number of transmitted founder viruses, unlike another C1-specific mAb (A32 IgG) (Santra et al., 2015). CH54 IgG and A32 IgG bind overlapping but distinct epitopes and have different ADCC breadth profiles (Bonsignori et al., 2012), which may explain their differing abilities to limit transmitted founder variants. By contrast, all CH31 bnAbs protected against rectal exposure, and the most potent neutralizing variant (CH31 IgG) was the most beneficial whether administered locally or systemically. Thus, the results from both the *ex vivo* and *in vivo* models were highly consistent and extend previous NHP findings indicating that bnAbs likely confer the most effective Ab-mediated protection (Burton et al., 2011, Dugast et al., 2014). However, a low dose intrarectal challenge model (Gautam et al., 2016) could be of interest to ascertain any protective activity of nnAbs individually or in combination.

We also compared the protective capacity of IgA2 mAb isoforms to their IgG1 counterparts following vaginal and rectal challenge. IgA2 can engage FcαR1 to mediate phagocytosis and ADCC *via* monocytes, macrophages and neutrophils, and dimeric forms can aggregate HIV-1 virions (Woof and Russell, 2011) but Env-specific IgA2 cannot mediate transcytosis of infectious virions *via* FcRn. We observed no impact of any 7b2 mAb variants on vaginal explant infectivity, despite differences in their *in vitro* functional profiles. Both 7b2 and CH31 IgG1s mediate phagocytosis and capture virions, but CH31 IgG captures more infectious virions (Liu et al., 2014), neutralizes HIV-1, and protects better in vaginal and rectal transmission models. The inferior protection afforded by CH31 mIgA2 compared to CH31 IgG1 corresponded with a marked loss in neutralization potency. Interestingly, the CH31 dimeric IgA2 isoforms, which capture virions better than the mIgA2 and can also aggregate virions, protected against intrarectal challenge better than their neutralization potencies would have predicted. A similar observation was made in the *ex vivo* vaginal challenge model. These results suggest that infectious virion capture and aggregation activities may augment modest, but not absent, neutralizing ability.

The protective capacity of other IgG and IgA isoforms of neutralizing mAbs has also been described. Unlike CH31, isotype switching to IgA2 improved 2F5 epitope binding, inhibition of transcytosis and maintained neutralization potency (Tudor et al., 2012). By contrast, monomeric and dimeric IgA1 variants showed inferior protection compared to 2F5 IgG in several models, including *in vivo* vaginal SHIV challenge (Klein et al., 2013b, Shen et al., 2010, Wolbank et al., 2003), despite the better virus aggregation abilities of 2F5 dIgA2. The compact *versus* flexible hinge region of IgA2 and IgA1, respectively, likely preserves the ability of the 2F5 Fv arms to protrude into the viral membrane, bind MPER and neutralize. Comparison of rectally instilled dIgA1, dIgA2, and IgG1 versions of the V3 mAb HGN194, with similar neutralizing potencies, showed that the dIgA1 provided the best protection against intrarectal SHIV challenge and protection correlated with virus capture and inhibition of virus transcytosis (Watkins et al., 2013). Interestingly, the combination of the dIgA2 and IgG1 delivered IV, synergized to provide complete protection, unlike each regimen on its own (Sholukh et al., 2015). Given the differences in structural architecture, FcR utilization and distribution of IgA Abs, the protective capability of this isotype in mucosal HIV-1 infection cannot be easily extrapolated by studying IgG mAbs, or a few mAb isotype variants. Dimeric IgA structures may enable additional antiviral functions but these gains may be at the expense of neutralization potency depending on the structural constraints associated with engaging a particular epitope.

Antibody-mediated protection is the result of a complicated interplay between the Ab, HIV, the immune system and other host factors. As such, measurements of individual Ab-mediated effects on HIV-1, be it augmentation of virus trafficking or inhibition by ADCC, cannot recapitulate the overall impact of an Ab on preventing infection. Thus, we utilized two mucosal models of HIV-1 transmission and functionally modified mAb variants to identify relevant, protective Ab properties, with an emphasis on the first few days of infection. Our results extend previous findings suggesting that extra-neutralization functions may augment the protective efficacy of bnAbs, but these functions alone are insufficient against early stages of infection (Dugast et al., 2014, Burton et al., 2011, Hessell et al., 2009b, Sholukh et al., 2015, Hessell et al., 2007). These are important implications in light of recent NHP studies suggesting that HIV-1 may spread to distal sites within 24 h of vaginal exposure, and that protection by bnAbs includes systemic clearance of these small, distal foci of infection, before plasma viral RNA is detectable (Barouch et al., 2016, Liu et al., 2016). Viral dissemination from the rectal mucosa may even be faster (Mcelrath et al., 2013, Miyake et al., 2006, Poles et al., 2001, Ribeiro Dos Santos et al., 2011), allowing less time to recruit effector cells from the periphery to contain the local infection.

Current HIV vaccine strategies for preventing mucosal infection focus primarily on the induction of bnAbs or V1 V2 IgG Abs with other antiviral functions. Vaccine efficacy trial outcomes require years of waiting but *in vivo* NHP and *ex vivo* human mucosal challenge models presented here can accelerate mechanistic insights and identify the most promising Ab features for mucosal protection. We found that IgA isoforms, typically associated with mucosal defense, were not inherently better than IgG1 for HIV-1 protection, and that the most important Ab function was neutralization capacity. Thus, defining strategies to provide potent Ab defense early with induction or passive infusion of Abs with broadly-neutralizing activities should remain the highest priority, but secondary antiviral functions mediated by bnAbs and nnAbs, such as ADCC and viral aggregation, may still have benefit if initial and rapid containment is not feasible.

## Conflicts of Interest

Dr. Barton F. Haynes has applied for patents for the CH31 antibody. Otherwise, there are no potential conflicts of interest to declare for any of the authors.

## Funding

Research reported in this publication was supported by funds from the Mucosal Immunology Group (BMGF OPP1099507), the Collaboration for AIDS Vaccine Discovery Grants from the Bill and Melinda Gates Foundation (OPP1040758; OPP1032144; OPP1032325; OPP1033098), and from the National Institute of Allergy and Infectious Diseases of the National Institutes of Health under award numbers UM1 AI068618 (Laboratory Center: HIV Vaccine Trials Network), UM1 AI069481 (Seattle-Lausanne-Kampala CTU), U19 AI067854 (the Center for HIV/AIDS Vaccine Immunology); UM1 AI100645 (Duke Center for HIV/AIDS Vaccine Immunology - Immunogen Discovery) and P30 AI027767 (Birmingham Center for AIDS Research, Virology Core).

The funders had no role in study design, data collection and analysis, decision to publish, or preparation of the manuscript. The content is solely the responsibility of the authors and does not necessarily represent the official views of the Bill and Melinda Gates Foundation or the National Institutes of Health.

## Figures and Tables

**Table 1:** Number of transmitted/founder viruses.

CH38 mIgA2	T/F variants	SGA sequences per animal	CH54-IgG_AAA	T/F variants	SGA sequences per animal
5731	–	Uninfected	5641	1	32
5691	3	26	5653	2	38
5689	3	27	5652	3	27
5688	4	35	5661	5	30
5708	8	33	5649	6	32
5715	> 11	34	5657	8	32
			5655	11	32
			5647	16	31
Median # T/F	3.5^a^		Median # T/F	3.5^a^	

Pavilizumab mIgA2 (control)	T/F variants	SGA sequences per animal	Pavilizumab IgG (control)	T/F variants	SGA sequences per animal
5711	3	28	5656	1	35
5614	3	29	5659	1	31
5640	3	29	5646	3	26
5637	4	31	5660	5	33
5721	4	25	5654	5	32
5722	> 10	25	5645	6	34
			5658	7	43
			5651	11	35
Median # T/F	5^a^		Median # T/F	5.5^a^	

a The difference in T/F variants between groups of monkeys treated with experimental antibodies and control antibodies did not achieve statistical significance.

## Appendix A Supplementary Data

Supplementary Material Image 1
