# Supplementary material for: Neutralization Takes Precedence Over IgG or IgA Isotype-related Functions in Mucosal HIV-1 Antibody-mediated Protection
Source: eBioMedicine. 2016 Nov 21;14:97–111. doi: 10.1016/j.ebiom.2016.11.024 (PMC5161443; doi:10.1016/j.ebiom.2016.11.024)
Supplement: Supplementary file 1 — Supplementary Material [file mmc1.pdf]

Supplemental Material

Supplemental Methods

Secreted NanoLuc™ reporter viruses

Previously, we described the construction of a *Renilla* luciferase (LucR) expressing, replication-competent HIV-1 proviral DNA (designated pNL-LucR.T2A), as well as several derivative LucR.T2A proviruses in which the ectodomain of gp160/Env is encoded by heterologous HIV-1 strain *env* sequences (deCamp et al., 2014, Edmonds et al., 2010, Montefiori et al., 2012, Ochsenbauer et al., 2012) (collectively referred to as pNL-LucR.T2A-Env.ecto proviral plasmids). We modified this approach by replacing the LucR coding region within the bicistronic LucR.T2A-nef fragment in pNL-LucR.T2A-Env.ecto proviral plasmids with the coding region for the secreted version of NanoLuc Luciferase (sNLuc) as present in the pNL1.3 (*secNluc*) vector (cat. #N1021, obtained from Promega, Madison, WI). A fragment flanked by *NheI – XbaI* restriction sites was synthesized in which the sNLuc stop codon was removed and the coding sequence appended with that of the T2A ribosomes-skipping peptide, essentially as described for the *NheI*-LucR.T2A-*Xba* fragment reported in (Edmonds et al., 2010); the resulting double-stranded DNA *NheI*-sNLuc.T2A-*Xba* fragment was subcloned into a cloning vector, then was excised with *NheI/XbaI* and inserted into equally digested pNL-LucR.T2A-Env.ecto proviral plasmids encoding the Env ectodomain of either HIV-1$_{Bal.26}$ (DQ318211.1) or HIV-1$_{JR\text{-}CSF}$ (M38429.1:6236.8782), resulting in pNL-sNLuc.T2A-Bal.26.ecto and pNL-sNLuc.T2A-JRCSF.ecto, respectively. In addition, an *env*-minus version was generated (pNL-sNLuc.T2A-mssD-Ala698, referred to herein as pNL-sNLuc.T2A-mssD) to provide a non-infectious control similarly to what we have previously generated and tested in the pNL-LucR.T2A backbone context: Briefly, we had previously reported (Edmonds et al., 2010) a cloning intermediate “shuttle vector” (pSP72.mss), designed with the viral EcoRI (NL4-3

nt5743) to XhoI (NL4-3 nt 8887) fragment from pNL4-3 into which unique silent restrictions sites had been introduced for cloning of heterologous *env* sequences, including the silent BstBI site (at nt 8301–8306 in the MSD coding region and used to insert heterologous *env* ectodomain coding regions) as well as a silent AgeI site (*env* nt 750-754) and silent PstI site (*env* nt 1245-1251). To facilitate screening for correct insertion of additional heterologous *env* ectodomain sequences, we previously cut pSP72.mss with AgeI/PstI, filled in the 5' overhang and removed the 3' overhang, respectively, and religated. In the resulting pSP72mssD, this effectively deleted NL4-3 *env* nt 755 through 1249, encoding Env aa 252-417, and replaced them with a Glycine codon. The EcoRI to BstBI fragment from pSP72mssD was then cloned back into pNL-LucR.T2A to generate pNL-LucR.T2A-mssD-Ala698. This proviral plasmid derivative does not only serve to facilitate cloning, but upon transfection generates HIV-1 virions which lack any infectivity in TZM-bl, T cell lines or PBMC (CO and JJ, unpublished). We then replaced the NheI-LucR.T2A-XbaI fragment with NheI-sNLuc.T2A-XbaI to generate plasmid pNL-sNLuc.T2A-mssD-Ala698 (referred to as pNL-sNLuc.T2A-mssD).

Preparation and titering of HIV-1 snLuc reporter viruses were done as previously described (Edmonds et al., 2010). In brief, generation of reporter viruses by transfection of proviral DNA into 293T/17 cells (ATCC, Manassas, VA) using Lipofectamine 2000, was done according to the manufacturer's protocol (Thermo-Fisher, Grand Island, NY). Viral supernatants were harvested 60 h post-transfection, clarified at 1200 × g for 10 min, and frozen at −70°C. Virus stocks were analyzed for snLuc expression using Nano-glo luciferase (Promega, Madison, WI) and were titered on sub-confluent TZM-bl cells (Wei et al., 2002) (obtained through the NIH AIDS Reagent Program, Division of AIDS, NIAID, as contributed by Dr. John C. Kappes, Dr. Xiaoyun Wu and Tranzyme Inc.). Virus was diluted in DMEM supplemented with 1% FBS and 40 µg/ml DEAE-Dextran and added to cells for 4

h. Growth medium (DMEM, 10% FBS, Pen/Strep, glutamine) were added to the cells and incubated for 48 hours. Cell monolayers were fixed with (0.8% glutaraldehyde, 2.2% Formaldehyde in DPBS) for 8 minutes and stained for β-galactosidase expression (4 mM potassium ferricyanide, 4mM potassium ferrocyanide, 400 μg/ml magnesium chloride, 400 μg/ml X-gal in DPBS) for 2 hours. Titer was calculated by counting “Blue” β-gal expressing cells.

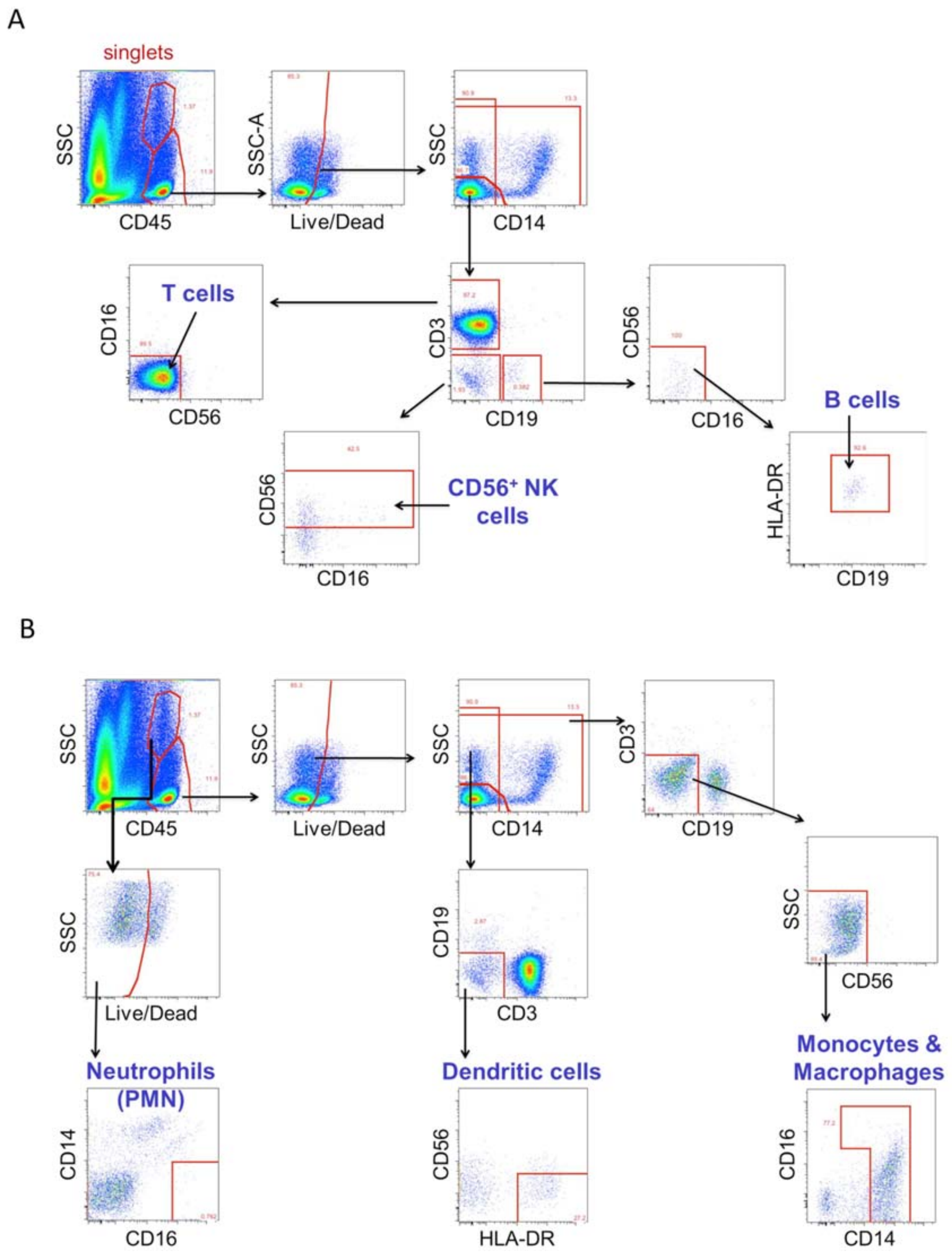

Figure S1. Representative gating scheme for the identification of major leukocyte populations from collagenase-digested human vaginal tissue. (A) CD45 bright leukocytes of low to intermediate side scatter (SSC) are first identified from the cell population, excluded of dead cells and then divided into 3 population: CD14- lymphocytes,

all CD14- leukocytes and non-lymphocytes. From the CD14- lymphocyte population, T cells (CD3+, CD16-, CD56-), B cells (CD3-, CD56-, CD16-, HLA-DR+) and CD56+ NK cells (CD3-, CD19-) are delineated. (B) CD45 dim, high SSC granulocytes are identified from the cell population, excluded of dead cells and gated on CD14 and CD16 to distinguish neutrophils (high SSC, CD14-, CD16+). From the all CD14- leukocytes population, dendritic cells (lineage negative, HLA-DR+) are identified. From the non-lymphocyte population, cells expressing lineage markers CD3, CD19 and CD56 are excluded and macrophages and monocytes (including classical and non-classical monocytes) are defined by CD14 and CD16 expression.

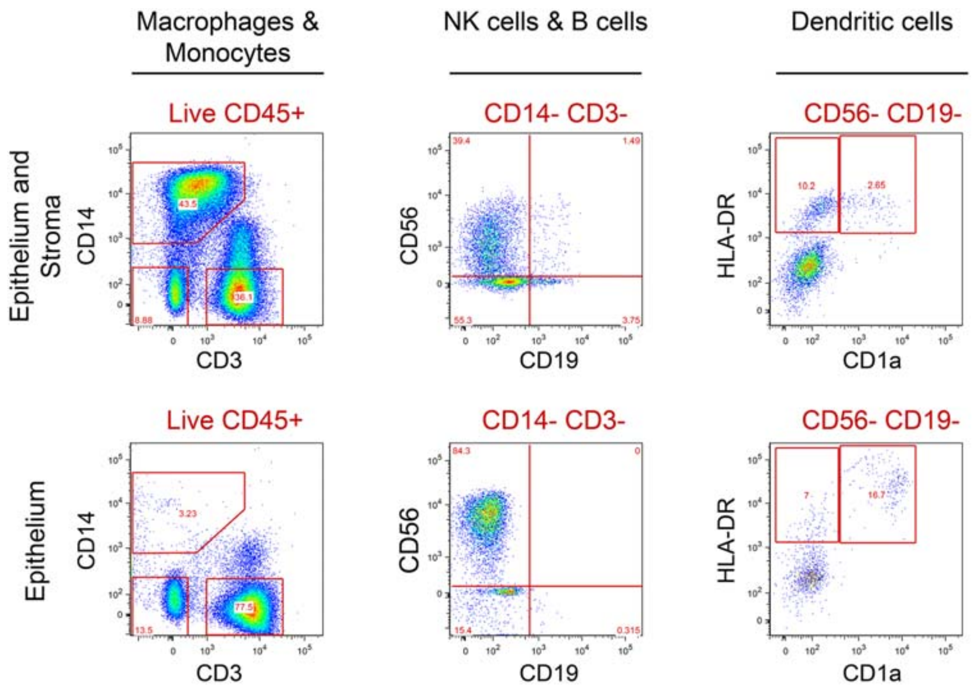

Figure S2. Differential distribution of leukocytes between stromal and epithelial compartments of human vaginal tissue. Representative FACS plots from three matched donors show the relative proportions of potential FcR-bearing leukocyte populations found in single cell suspensions isolated from whole vaginal tissue (top panel) or separated vaginal epithelial sheets (bottom panel). Macrophages and monocytes (Hi SSC, CD45+, CD14+) and B-cells (CD45+, CD3-, CD56-, CD14-, CD19+) are predominantly sub-epithelial. Intraepithelial dendritic cells are primarily Langerhans cells (lineage-, HLA-DR+, CD1a+). NK cells (CD45+, CD3-, CD14-, CD56+), can be found in both regions.

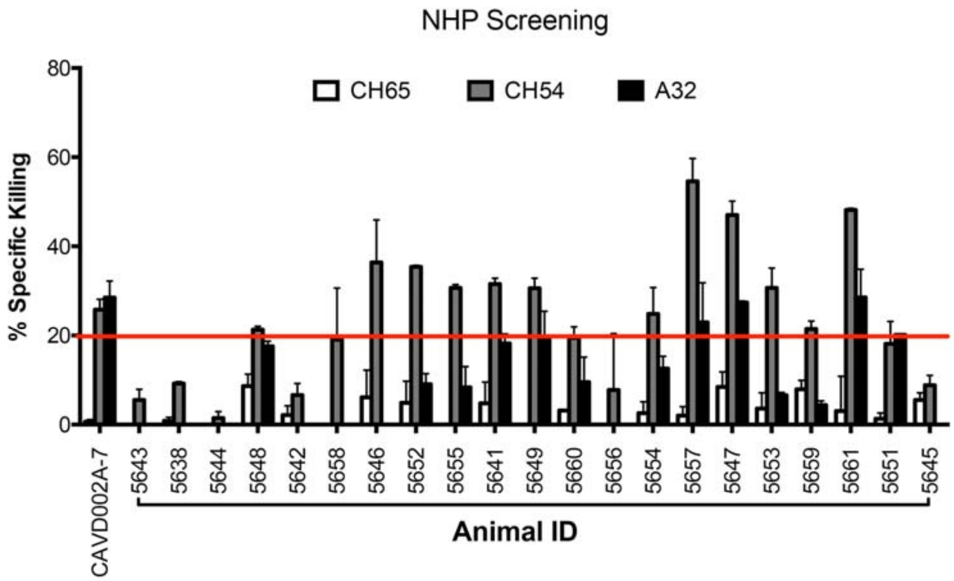

ADCC with BAL-IMC infected CEM.NKR Target cells.

Figure S3. Selection of macaques for CH54 IgG protection studies via in vitro ADCC screening using a reporter HIV-1 BaL-IMC. The purpose of this experiment was to evaluate the ability of the mAb IgG to functionally engage the Fc-bearing NHP NK cells. The impaired Nef, but not Vpu, function associated with the IMC construct design (Edmonds et al., 2010) delays downregulation of the CD4 (Alberti et al., 2015) in infected cells. This results in a population of target cells (36 h post-infection) with heterogenous downregulation CD4, which allows for binding of the C1 specific mAbs to these infected cells. The anti-flu CH65 IgG was used as the negative control. The human CAVD002A-7 PBMCs were used as the control for the effector population. Animals with greater than 20% specific killing, indicated by the red line, were selected for CH54 IgG protection studies.

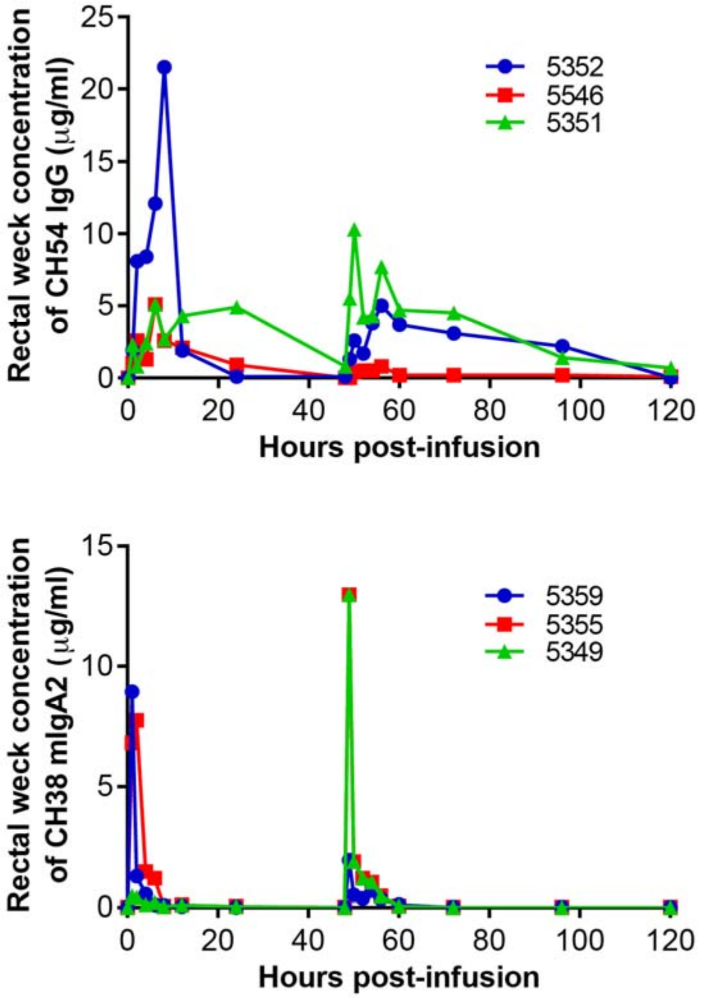

Figure S4. Concentration of CH54 IgG and CH38 mIgA2 in rectal secretions following systemic infusions. Rhesus monkeys were passively infused with CH54 IgG (top) or CH38 IgA2 (bottom) mAbs at 0 and 48 h. Rectal secretions were collected at indicated time points and mAb concentrations were determined by a binding assay.

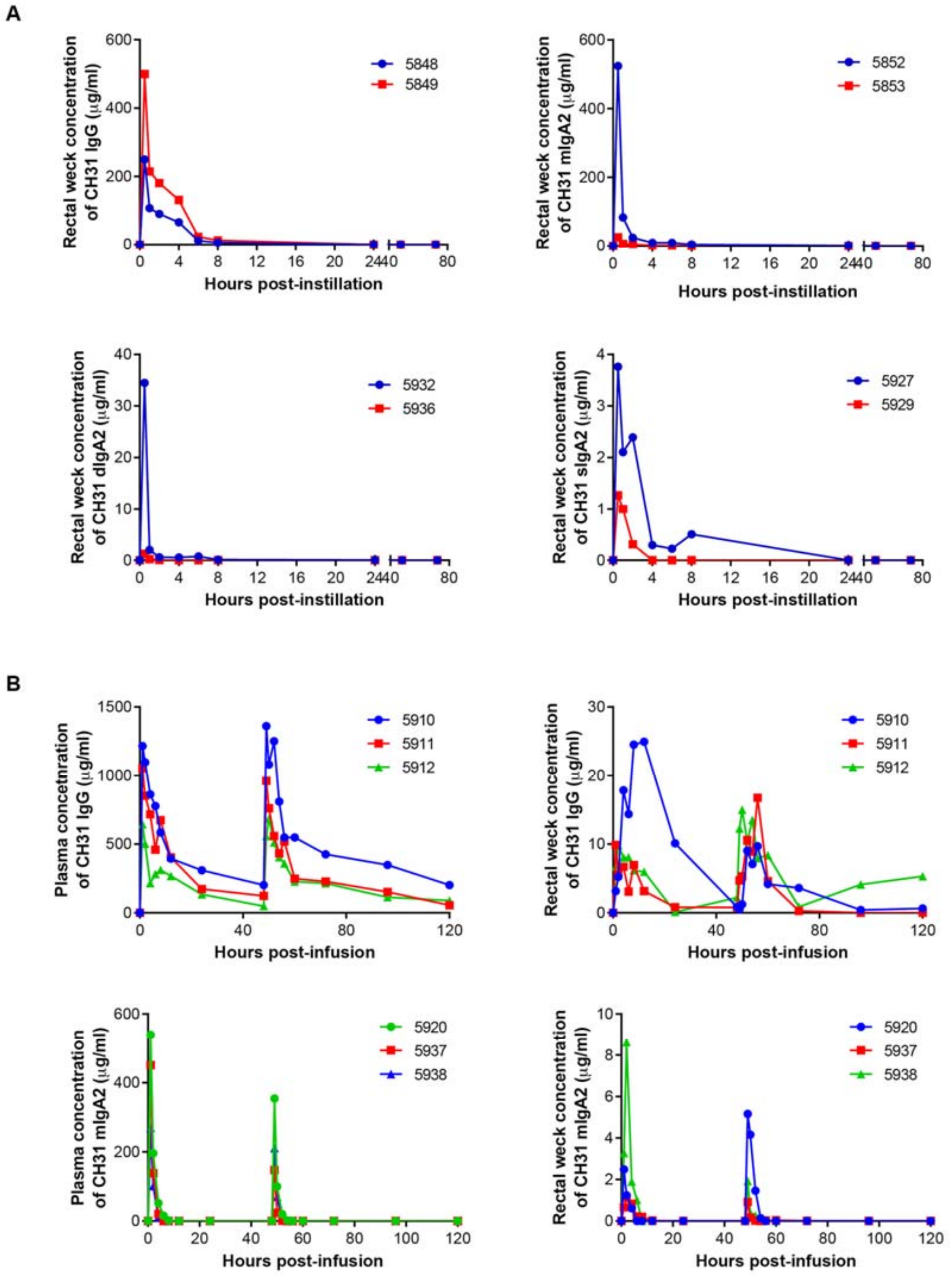

Figure S5. Monoclonal antibody concentrations in plasma and rectal secretions of rhesus monkeys following monoclonal antibody treatments. (A) Rhesus monkeys were treated by rectal instillation with four different isotypes of CH31 monoclonal antibody including CH31 IgG, CH31 mIgA2, CH31 dIgA2 and CH31 sIgA2. Post-instillation rectal

secretions were collected at indicated time points and concentrations of instilled antibodies were determined by a binding assay. (B) Monkeys were passively infused with either CH31 IgG or CH31 mIgA2 at 0 and 48 h. Plasma and rectal washes were collected at time points indicated. Post-infusion concentrations of antibodies in plasma and rectal washes were determined by a binding assay.

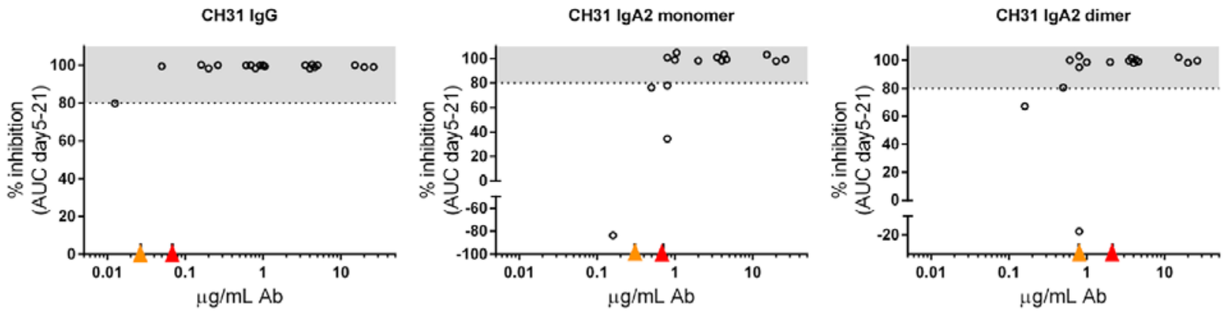

Figure S6. Determination of *ex vivo* lowest effective doses (LED80) for CH31 mAbs against snLuc.HIV-1$_{\text{Bal26}}$. The area under the kinetic luciferase assay curves depicted in Figure 6A were calculated from Day 5-21 for CH31 mAbs and their matched positive (virus only and isotype controls) and negative infection controls (delta env or AZT)**.** The latter values were used for background subtraction. Percent inhibitions were calculated within each donor matched experiment as follows: (1 - AUC at × µg/mL Ab - background AUC) /(Average AUC for positive infection controls - background AUC) × 100%. *In vitro* neutralization IC50 and IC80 values are marked on the x-axis for reference with orange and red arrowheads, respectively, for reference. The *ex vivo* efficacy cutoff was set at 80% inhibition and the lowest mAb concentration that met this threshold in the majority of donor tissues was designated as the *ex vivo* LED80 for a given mAb (see Figure 6).

Table S1. Antibodies used in staining panels to determine all major blood leukocyte populations and Fc receptors.

Marker	Fluorophore	Supplier	Clone	RRID	Panel*
CD45	V450	BD Biosciences	2D1	AB_1645755	1, 2
CD3	AlexaFluor700	BD Biosciences	UCHT1	AB_396952	1, 2
CD14	APC H7	BD Biosciences	MφP9	AB_1645726	1, 2
CD19	FITC	BD Biosciences	HIB19	AB_395812	1
CD1a	AlexaFluor647	Dendritics	201B5.08	AB_1148714	1
CD56	PE-Cy7	BD Biosciences	NCAM16.2	AB_399970	1
HLA-DR	ECD	Beckman Coulter	Immu-357	AB_10643231	1
CD3	BV650	BioLegend	OKT3	AB_11126748	2
CD19	BV605	BD Biosciences	SJ25C1		2
CD56	FITC	BD Biosciences	NCAM16.2	AB_400121	2
HLA-DR	BV711	BioLegend	L243	AB_11218794	2
CD16	PE-Cy7	BD Biosciences	3G8	AB_1727433	2
CD32	PerCP-eFluor710	eBioscience	6C4	AB_11218874	2
CD64	APC	BD Biosciences	10.1	AB_2536514	2
CD89	PE	BD Biosciences	A59	AB_396037	2

* Panel 1 relates to Figure S1 and panel 2 relates to Figures 1 and S2.

Table S2. Neutralization potency of CH31 mAbs.

	IC50, μg/ml				
	CH31				2F5
Viruses	sIgA2	Dimeric IgA2	Monomeric IgA2	IgG1	IgG1
**SHIV-BAL-P4**	**18.464**	**14.516**	**2.184**	**5.016**	**3.145**
**SHIV-SF162P3**	**27.673**	**18.11**	**7.827**	**0.841**	**0.841**
**BAL26**	**0.434**	**0.796**	**0.306**	**<0.023**	**1.165**
**MuLV**	>50	>50	>50	>50	>50

	IC80, μg/ml				
	CH31				2F5
Viruses	sIgA2	Dimeric IgA2	Monomeric IgA2	IgG1	IgG1
**SHIV-BAL-P4**	>50	>50	**7.784**	>50	**16.354**
**SHIV-SF162P3**	>50	>50	**24.28**	**2.434**	**0.841**
**BAL26**	**1.227**	**2.142**	**0.706**	**0.068**	**6.166**
**MuLV**	>50	>50	>50	>50	>50

References:

ALBERTI, M. O., JONES, J. J., MIGLIETTA, R., DING, H. T., BAKSHI, R. K., EDMONDS, T. G., KAPPES, J. C. & OCHSENBAUER, C. 2015. Optimized Replicating Renilla Luciferase Reporter HIV-1 Utilizing Novel Internal Ribosome Entry Site Elements for Native Nef Expression and Function. *Aids Research and Human Retroviruses,* 31**,** 1278-1296.

DECAMP, A., HRABER, P., BAILER, R. T., SEAMAN, M. S., OCHSENBAUER, C., KAPPES, J., GOTTARDO, R., EDLEFSEN, P., SELF, S., TANG, H., GREENE, K., GAO, H., DANIELL, X., SARZOTTI-KELSOE, M., GORNY, M. K., ZOLLA-PAZNER, S., LABRANCHE, C. C., MASCOLA, J. R., KORBER, B. T. & MONTEFIORI, D. C. 2014. Global panel of HIV-1 Env reference strains for standardized assessments of vaccine-elicited neutralizing antibodies. *J Virol,* 88**,** 2489-507.

EDMONDS, T. G., DING, H., YUAN, X., WEI, Q., SMITH, K. S., CONWAY, J. A., WIECZOREK, L., BROWN, B., POLONIS, V., WEST, J. T., MONTEFIORI, D. C., KAPPES, J. C. & OCHSENBAUER, C. 2010. Replication competent molecular clones of HIV-1 expressing Renilla luciferase facilitate the analysis of antibody inhibition in PBMC. *Virology,* 408**,** 1-13.

MONTEFIORI, D. C., KARNASUTA, C., HUANG, Y., AHMED, H., GILBERT, P., DE SOUZA, M. S., MCLINDEN, R., TOVANABUTRA, S., LAURENCE-CHENINE, A., SANDERS-BUELL, E., MOODY, M. A., BONSIGNORI, M., OCHSENBAUER, C.,

KAPPES, J., TANG, H., GREENE, K., GAO, H., LABRANCHE, C. C., ANDREWS, C., POLONIS, V. R., RERKS-NGARM, S., PITISUTTITHUM, P., NITAYAPHAN, S., KAEWKUNGWAL, J., SELF, S. G., BERMAN, P. W., FRANCIS, D., SINANGIL, F., LEE, C., TARTAGLIA, J., ROBB, M. L., HAYNES, B. F., MICHAEL, N. L. & KIM, J. H. 2012. Magnitude and breadth of the neutralizing antibody response in the RV144 and Vax003 HIV-1 vaccine efficacy trials. *J Infect Dis,* 206**,** 431-41.

OCHSENBAUER, C., EDMONDS, T. G., DING, H., KEELE, B. F., DECKER, J., SALAZAR, M. G., SALAZAR-GONZALEZ, J. F., SHATTOCK, R., HAYNES, B. F., SHAW, G. M., HAHN, B. H. & KAPPES, J. C. 2012. Generation of transmitted/founder HIV-1 infectious molecular clones and characterization of their replication capacity in CD4 T lymphocytes and monocyte-derived macrophages. *J Virol,* 86**,** 2715-28.

WEI, X., DECKER, J. M., LIU, H., ZHANG, Z., ARANI, R. B., KILBY, J. M., SAAG, M. S., WU, X., SHAW, G. M. & KAPPES, J. C. 2002. Emergence of resistant human immunodeficiency virus type 1 in patients receiving fusion inhibitor (T-20) monotherapy. *Antimicrob Agents Chemother,* 46**,** 1896-905.
